# Brief virtual nature exposure and acute stress: inconclusive evidence regarding stress reactivity, recovery, and emotion regulation

**DOI:** 10.1016/j.jenvp.2026.103181

**Published:** 2026-09

**Authors:** M.O. Steininger, J.P. Nitschke, L. Schenk, M.P. White, C. Lamm

**Affiliations:** a Department of Cognition, Emotion, and Methods in Psychology, Faculty of Psychology, University of Vienna, Vienna, Austria; b Department of Clinical & Health Psychology, Faculty of Psychology, University of Vienna, Vienna, Austria; c Cognitive Science Hub, University of Vienna, Vienna, Austria; d European Centre for Environment and Human Health, University of Exeter, UK; e Environment and Climate Research Hub, University of Vienna, Vienna, Austria

**Keywords:** Nature exposure, Stress, Acute stress, Stress reactivity, Stress recovery, Emotion regulation

## Abstract

Exposure to nature is widely considered to alter acute stress. Most research has focused on nature’s ability to facilitate stress recovery, while little is known about its effects on initial reactivity to a stressor and associated emotion regulation strategies. Guided by Nature-Based Biopsychosocial Resilience Theory, we investigated whether brief virtual nature exposure influences both stress reactivity and recovery, as well as stressor-specific appraisal and rumination. In a randomized controlled study, 164 healthy adults watched brief, two-dimensional virtual nature or indoor scenes either before or after an acute laboratory stress task. We repeatedly measured self-reported stress, salivary cortisol, heart rate, and heart rate variability to assess stress reactivity and recovery, alongside stressor-specific appraisal and rumination, and collected retrospective ratings of the environments’ perceived restorativeness and helpfulness in dealing with the stressor. Participants retrospectively rated nature videos as more restorative and helpful in managing and recovering from the stressor than indoor videos (moderate-to-large effects). In contrast, none of the registered hypotheses about the positive effects of nature videos on immediate overall stress responses, stress reactivity, stress recovery, appraisal, or rumination were significant. However, equivalence tests indicated that small-to-moderate effects cannot be ruled out. The results highlight a potential disconnect between participants’ retrospective perceptions of nature’s capacity to help cope with a stressor and their immediate psychological and physiological reactions. Although virtual nature may represent an easily implementable intervention, our findings encourage a critical reflection and calibration of expectations regarding the effectiveness of brief simulated nature exposures for reducing acute stress.

## 1 Introduction

### 1.1 Nature, health and stress

A growing body of research demonstrates that contact with nature supports both physical and mental health (Frumkin et al., 2017; Markevych et al., 2017; Twohig-Bennett & Jones, 2018; Zhang et al., 2021). Various forms of nature contact, such as residing in green neighborhoods, visiting urban parks, or engaging in outdoor activities, have been linked with a broad spectrum of health benefits, from reduced risks of preterm birth to lower incidences of psychiatric disorders (Agay-Shay et al., 2014; Engemann et al., 2019; Wolf & Wohlfart, 2014). Altered stress responses are widely considered a central pathway through which nature may influence well-being (Hartig et al., 2014). Stress Reduction Theory (SRT) explicitly positions reduced stress as the foundational mechanism linking nature and health, proposing that exposure to non-threatening natural environments enhances recovery from stressful experiences (Ulrich et al., 1991).

Empirical evidence supports nature’s role in stress regulation. In a seminal study, and a recent multisite replication, participants who viewed natural versus urban scenes recovered more quickly from stress, as indicated by self-reported and physiological measures (Ulrich et al., 1991; Van Den Berg et al., 2026). Observational studies further suggest that neighborhood greenspace is associated with healthier patterns of cortisol secretion (Thompson et al., 2012) and living near environments with more nature is associated with lower chronic stress (Gidlow et al., 2016). Laboratory studies complement these findings, showing that simulated nature exposure facilitates recovery from experimentally induced acute stress, thus offering converging evidence and addressing some of the limitations inherent to observational research (Annerstedt et al., 2013; Liszio et al., 2018; Thoma et al., 2013).

Despite these promising findings, recent research questions the consistency and temporal dynamics of nature’s effects on different components of stress. On the one hand, recent studies of nature’s impact on acute laboratory stress responses report inconclusive results. For example, exposure to a virtual green environment, compared with an office setting, failed to facilitate recovery from an acute stressor, as no changes in cortisol levels or arterial pressure related to the green environment were detected (Yin et al., 2022). Similarly, other studies reported that virtual nature did not promote stress recovery when assessed via cortisol or heart rate (HR), and revealed inconsistent effects on heart rate variability (HRV) or skin conductance (Knaust et al., 2022; Michels et al., 2021; Sun et al., 2023). On the other hand, a key limitation of earlier work is its narrow focus on stress recovery. While this focus aligns with the traditional emphasis on nature’s role in accelerating recovery processes (Ulrich et al., 1991), it neglects the broader temporal structure of how nature may act on the stress response.

### 1.2 Nature, stress reactivity, and recovery

More recent theoretical frameworks have broadened their scope and highlight multiple stages of the stress response when discussing nature-health associations. Nature-Based Biopsychosocial Resilience Theory (NBRT; White et al., 2023), for instance, proposes that nature may influence stress across three stages in the stress-response-recovery cycle. First, nature may reduce stress exposure by mitigating the intensity of stressors, such as when trees or hedges reduce noise pollution from roads for nearby households. This is referred to as *preventive resilience* and is the focus of many Nature-based Solutions (Castellar et al., 2021), but not the current work. Second, and more relevant here, it can attenuate acute stress reactivity, including hypothalamic-pituitary-adrenal (HPA) axis activation or sympathetic arousal. In NBRT, this capacity is referred to as *response resilience*. For example, nature (vs. urban) walks, or watching nature (vs. control) videos, *prior* to a stressor can reduce amygdala activity (Sudimac et al., 2022) and autonomic arousal (Wells, 2005) during subsequent stress tasks. Third, and consistent with SRT (Ulrich et al., 1991), nature may promote recovery by supporting a faster or more complete return to homeostasis after stress, a process termed *recovery resilience*.

Previous laboratory research has predominantly focused on recovery resilience, typically testing whether nature exposure succeeding a stressor accelerates the return to baseline (e.g., Annerstedt et al., 2013; Sun et al., 2023; Ulrich et al., 1991; Yin et al., 2020). Far fewer studies have tested whether nature exposure before a stressor enhances adaptive reactions to stress, as proposed by NBRT’s concept of response resilience (e.g., Brown et al., 2013; Michels et al., 2021). Moreover, to our knowledge, no study has explicitly compared reactivity and recovery phases within a design capable of disentangling these processes. The present study therefore aimed to investigate how brief virtual nature exposure supports both response and recovery resilience.

### 1.3 Nature and stressor-specific emotion regulation

NBRT argues that resilience processes involve biological, psychological, and social resources. Accordingly, understanding response and recovery resilience requires examining not only immediate physiological or hormonal responses at the biological level but also adaptive or maladaptive antecedent- and response-focused emotion regulation strategies at the psychological and social levels (Gross, 1998, 2001; Lewis et al., 2018; White et al., 2023). Antecedent-focused strategies are engaged at both the preventive stage and the initial reaction to a stressor and include processes such as avoidance or primary and secondary appraisal. Primary appraisal refers to evaluations of the perceived threat or challenge of a stressful situation, and secondary appraisal refers to one’s perceived ability to manage it (Bandura, 1977; Gaab et al., 2005). In the current context, prior nature exposure may be associated with a stressor seeming less daunting (primary appraisal) and one’s ability to cope being enhanced (secondary appraisal). Response-focused strategies occur after the stress response has been initiated and include adaptive and maladaptive regulatory efforts such as acceptance, suppression, or rumination (S. L. Edwards et al., 2003; Gross, 1998). These strategies align with the more traditionally researched recovery phase after the initial peak stress response has been experienced.

Importantly, examining changes in both antecedent-focused (e.g. appraisal; response resilience) and response-focused (e.g. rumination; recovery resilience) strategies over time may offer a valuable window into how nature exposure shapes the overall stress cycle (Bratman et al., 2021). However, these processes remain understudied within the nature-stress literature. Although previous research suggests that nature contact may generally attenuate self-reported and neural correlates of global ruminative tendencies (Bratman et al., 2015; Lopes et al., 2020), its influence on stressor-specific appraisal or rumination remains unclear.

### 1.4 Study objectives

Given the shortcomings outlined above, this study aimed to clarify how exposure to nature influences stress reactivity and recovery, as well as associated emotion regulation strategies during acute stress. To isolate nature’s distinct effects on these processes, we systematically manipulated the timing of nature exposure relative to an acute laboratory stressor. Throughout the experiment, we repeatedly assessed self-reported, autonomic, and hormonal indicators of immediate stress responses, alongside antecedent- and response-focused stressor-specific emotion regulation strategies. Participants were randomly assigned to one of four experimental conditions, each involving exposure to either a brief virtual nature scene or an indoor scene acting as a control condition, presented either before or after the stressor (resulting in the following groups: nature pre/post stressor, control pre/post stressor). We tested five registered hypotheses.

To begin, we expected that nature exposure (irrespective of its timing relative to the stressor) would reduce overall stress responses, reflected in a smaller area under the stress curve (H1). This could occur via lower initial reactivity to a stressor following nature exposure (H2), or, as more traditionally researched, via quicker recovery through nature exposure after the stressor (H3). To investigate underlying psychological resilience-related processes, we focused on appraisal and rumination mechanisms. Specifically, we predicted that nature exposure prior to the stressor would lower primary (threat and challenge) and increase secondary (control and competence) appraisals (H4). In contrast, nature exposure after the stressor was expected to increase positive and reduce negative stressor-specific ruminative thoughts (H5). Lastly, we registered that virtual nature scenes would be perceived as more restorative and helpful in coping with the stressor than indoor scenes (H6).

## 2 Methods

### 2.1 Participants

The study was registered after data collection on 21 February 2025 (https://osf.io/w7b8x). Thus, data collection had been completed at the time of registration. Importantly, no inspection or analysis of the data was carried out prior to registration. Accordingly, while the registration does not constitute a fully prospective preregistration, it constrained analytic decision-making prior to data access and contributes to transparency in analyses and reporting. The study was approved by the Ethics Committee of the University of Vienna (EK-Nr. 00928), and conducted in line with the seventh revision of the Declaration of Helsinki (2013). Based on an a-priori power analysis using prior studies on nature and acute stress (Annerstedt et al., 2013; Liszio et al., 2018; Thoma et al., 2013), we registered a sample size of N = 166 participants. Using a moderate effect size (f = 0.27), α = .05, and 1-β = 0.8 for a one-way four-group ANOVA resulted in a required sample size of N = 156. To account for potential exclusions, we recruited 167 participants (see Supplementary Methods for details). The sample included healthy participants aged 18-38 who identified as male or female and met standard eligibility criteria for studies assessing hormonal and autonomic responses to acute stress (see Supplementary Methods for details). Three participants were excluded (language difficulties, exceeding age limit, early termination of acute stress task), resulting in a final sample of 83 female and 81 male participants (Age ± SD = 24.59 ± 4.43). Participants received reimbursements of 20-30€ depending on session duration.

### 2.2 Procedure

**Fig. 1:**
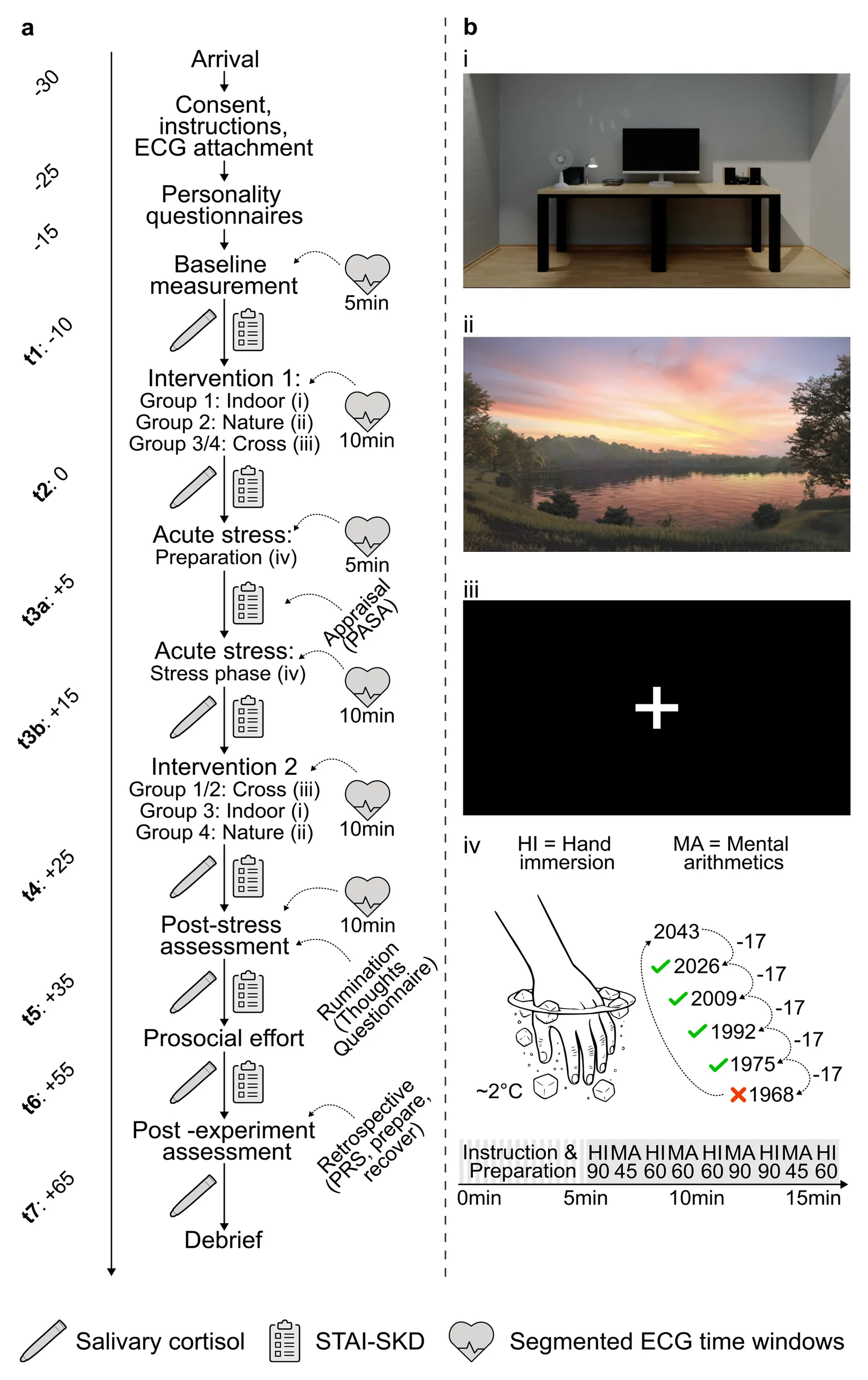
Study procedure and design. **(a)** Study procedure showing the sequence of phases, their timing relative to the acute stressor, and the measurements collected. Salivary cortisol and the State Trait Anxiety Inventory (short form; STAI-SKD) were assessed at seven time points, and ECG was segmented into six intervals of 5 or 10 min. **(b)** Experimental materials. Panels *(i-ii)* display the brief indoor *(i)* and nature *(ii)* video environments, consisting of 10-min videos with accompanying soundscapes. Panel *(iii)* shows the control task (fixation cross). Panel *(iv)* depicts the Maastricht Acute Stress Test (MAST). The lower part of panel *(iv)* illustrates the MAST structure: a 5-min instruction and preparation phase, followed by a 10-min acute stress phase with alternating blocks of hand immersions (HI) in ice water (~2 °C) and mental arithmetic (MA) tasks, in which participants continuously and rapidly counted backwards in steps of 17 from a high starting number. Errors resulted in negative feedback and required participants to restart the mental arithmetic task from the initial number. Both block types had varying, fixed durations (in seconds). During the MAST, participants were visibly monitored by a confederate who provided continuous neutral or negative feedback on participants performance. Participants were also deceived into believing their performance would be videotaped and analyzed for verbal and bodily reactions. Study groups differed in timing and type of video exposure relative to the MAST: Groups 1 and 2 viewed videos before the stressor (pre), while Groups 3 and 4 viewed videos after (post). Groups 1 and 3 were exposed to the indoor environment *(i)*, and Groups 2 and 4 to the nature environment *(ii)*.

The experimental procedure is illustrated in Fig. 1. All sessions took place in a secluded, private room without the presence of other participants or external distractions. In addition, all sessions were conducted between 2:00 and 7:00 p.m. to control for diurnal variations in cortisol levels (S. Edwards et al., 2001). To minimize confounding influences on cortisol responses, participants were instructed to abstain from smoking, alcohol, recreational drugs, medication, and physical exercise for 24 h, and from eating, drinking (except water), brushing teeth, or using chewing gum for 2 h before the session. Upon arrival, participants confirmed adherence to these requirements and provided written informed consent. They then received oral instructions, attached the electrocardiogram (ECG) device (with continuous recording beginning at this point), completed questionnaires (10 min), and underwent a 5-min baseline ECG recording while seated (t1). Participants then completed the first intervention (t2): viewing either a virtual indoor (group 1) or a virtual nature (group 2) environment, or a fixation cross (groups 3 and 4) for 10 min. All participants subsequently completed an acute stress task (t3; see below). This was followed by the second intervention (t4), in which groups 1 and 2 viewed a fixation cross, while the other groups watched a virtual indoor (group 3) or a virtual nature (group 4) environment for 10 min. Immediately afterward, participants completed a post-stress questionnaire related to the stress task (t5; 10 min), followed by a 20-min prosocial effort task (t6; not relevant to the present article). Lastly, they rewatched a 1.5-min version of their assigned environment, rated its perceived restorativeness, helpfulness to manage or recover from a hypothetical real-life stressful situation, and its perceived helpfulness in coping with the stressor experienced in the experiment (t7). Participants were then debriefed and compensated. Group sizes for groups 1 through 4 were 42, 41, 41, and 40 participants, respectively.

### 2.3 Virtual environments

The virtual environments were presented on a computer monitor with matching soundscapes delivered through headphones (t2/t4). Before exposure to the environments, participants were instructed to focus on both visual and auditory elements and to imagine being present in the environments, using written immersion scripts adapted from prior nature-based guided imagery studies (Coughlan et al., 2022; Nguyen & Brymer, 2018). The nature scene depicted a serene lakeside landscape with surrounding meadows, trees, and hills, progressing from sunrise to sunset, accompanied by natural sounds (e.g., wind, water, birds, insects; Fig. 1b(ii)). The scene consisted entirely of natural features and had a green view index (i.e., proportion of visible vegetated areas) of 0.372 (GVI; Aikoh et al., 2023; Aoki, 1991), with the remaining visual content comprising water and sky elements. The accompanying soundscape showed a peak-level of -6.46 dBFS, a root-mean-square (RMS) level of -34.34 dBFS, a dynamic range of 36.22 dB, a spectral centroid of 5008 Hz, and an acoustic diversity index (i.e., complexity and richness of the audio) of 1.67 (ADI; Villanueva-Rivera et al., 2011). The indoor condition featured an animated office room with a desk, computer, and supplies, paired with ambient indoor sounds (computer hum, fan noise, indistinct background chatter; Fig. 1b(i)). As intended during stimulus construction, this condition contained no natural visual elements, resulting in a GVI = 0.00, with no water or sky components. The accompanying soundscape showed a peak-level of −10.31 dBFS, an RMS of −32.61 dBFS, a dynamic range of 35.75 dB, a spectral centroid of 2793 Hz, and an ADI of 0.09. Overall, the nature scene was characterized by the presence of green, blue, and sky-related visual elements, whereas these were absent in the indoor condition. Acoustic parameters indicated similar peak and average sound levels across conditions, while the nature soundscape exhibited higher spectral brightness and greater acoustic diversity relative to the indoor soundscape. Further details on visual and acoustic property estimation are provided in the Supplementary Results. Both videos had been validated in prior studies (Smalley et al., 2023; Steininger et al., 2025). To ensure procedural consistency, participants not exposed to a virtual environment viewed a white fixation cross on a black background without sound (Fig. 1b(iii)). The groups differed in the timing and content of the virtual environment exposure, which occurred either before (t2 for groups 1 and 2) or after (t4 for groups 3 and 4) the acute stressor, showing indoor (groups 1 and 3) or nature environments (groups 2 and 4). Participants were stratified by gender and randomly assigned to one of the four groups. We fully randomized group order within each stratum and assigned participants sequentially based on study entry.

### 2.4 Acute stress

Acute stress (t3) was induced using the Maastricht Acute Stress Task (MAST; Smeets et al., 2012), a validated protocol eliciting psychosocial evaluative threat and unpredictability. The procedure included a 5-min preparation phase and a 10-min stress phase (Fig. 1b(iv)). A confederate in a white lab coat delivered instructions and deceived participants into believing they would be video-recorded and evaluated on their task performance, including their facial expressions and gestures. The stress phase consisted of five cold pressor trials (Fig. 1b(iv)) combined with an arithmetic challenge task. During cold pressor trials, participants sub-merged their dominant hand in ice-cold water (M = 2.36 °C, SD = 0.66) for up to 90 s each. Participants were told that the trial durations were randomly determined by a computer, enhancing unpredictability, although durations followed a fixed sequence (Fig. 1b(iv)). Following each immersion, participants completed a mental arithmetic task where they had to count backwards in steps of 17 from a high number, as quickly and accurately as possible (Fig. 1b(iv)). Errors resulted in negative feedback from the confederate and required participants to restart the mental arithmetic task from the initial number. The complete MAST was maximized to increase social-evaluative pressure.

### 2.5 Self-reported measures

Self-reported stress was repeatedly assessed using the short state-form of the State-Trait Anxiety Inventory in German (STAI-SKD; Englert et al., 2011) at seven timepoints (t1-t6) relative to stressor onset (−10, 0, 5, 15, 25, 35, and 55 min). The STAI-SKD provides a sum score based on five items. Anticipatory cognitive appraisal was measured with the Primary and Secondary Appraisal Scale (PASA; Gaab et al., 2005) between the preparation and stress phases of the MAST (t3a). The PASA consists of 16 items assessing either primary (perceived threat and challenge) or secondary (perceived control and competence) appraisal of the upcoming stressor. After the second intervention phase (t5), participants completed an adapted version of the Thoughts Questionnaire (S. L. Edwards et al., 2003), a post-event rumination measure capturing situation-specific positive and negative ruminative thoughts related to the acute stress phase. The questionnaire included 11 items targeting positive rumination (e.g., how well they handled the situation) and 16 items assessing negative rumination (e.g., that they made a fool of themselves). Participants rated how frequently they had engaged in these thoughts since the conclusion of the stressor. At the end of the experiment (t7), participants rated the perceived restorativeness of the virtual environment using an adapted version of the Perceived Restorativeness Scale (PRS), consisting of five items, which were summed to yield an overall restorativeness score (Berto, 2005; Hartig et al., 1997). Additionally, they rated two single items assessing whether they believed being in the respective environment would help them to (a) manage or (b) recover from a hypothetical real-life stressful event. Furthermore, participants provided single-item retrospective ratings assessing how much being exposed to the virtual environment helped them prepare for the MAST (groups 1 and 2) or recover from it (groups 3 and 4). Further details on the structure, content, and psychometric properties of the questionnaires are provided in the Supplementary Methods.

### 2.6 Autonomic measures

Heart rate (HR) and heart rate variability (HRV) were derived from ECG signals continuously recorded at 1.000 Hz using the Bittium Faros 180° device (Bittium Inc., Oulu, Finland) with three adhesive electrodes placed below the right and left clavicles and the left ribcage. ECG was recorded while participants were seated and segmented into six pre-defined non-overlapping time windows corresponding to the experimental phases. Baseline (t1) and stress anticipation (t3a) recordings lasted 5 min, while recordings during the interventions (t2/t4), acute stress (t3b), and post-stress questionnaires (t5) lasted 10 min. Data were preprocessed and analyzed in MATLAB R2023a (The MathWorks, Inc., 2023), using HRVTool (version 1.07; Vollmer, 2019, p. 1). We extracted RR intervals (i.e., the time between consecutive R-waves) and preprocessed the data semiautomatically. Artifacts (e.g., ectopic or missing R-peaks) were filtered using filter-based algorithms, automated pattern detection and return maps of relative RR intervals (Vollmer, 2014, 2017), followed by manual visual inspection and correction, whenever necessary. HR was derived from the RR intervals and expressed in beats per minute (bpm). HRV was quantified using the root mean square of successive differences (RMSSD), a time-domain measure indexing vagally mediated short-term heart beat peak variability (Shaffer & Ginsberg, 2017). Data from two participants were excluded from HR analyses (but retained for the other analyses) due to improperly attached electrodes resulting in signal quality insufficient for reliable heartbeat detection.

### 2.7 Cortisol measures

We collected salivary samples using Salivette® synthetic swabs (Sarstedt, Nümbrecht, Germany) at seven timepoints relative to stressor onset (−10, 0, 15, 25, 35, 55 and 65 min). Participants placed the swab in their mouth for at least 2 min and were instructed to avoid chewing or excessive movement. Samples were immediately frozen and stored at −25 °C until analysis at the Dresden LABService GmbH (Dresden, Germany). After thawing, samples were centrifuged at 3000 rpm for 5 min, which resulted in a clear supernatant of low viscosity. Salivary cortisol concentrations were measured using a commercially available chemiluminescence immunoassay with high sensitivity (Tecan - IBL International, Hamburg, Germany; catalogue number R62111). The intra- and interassay coefficients of variance for Cortisol assays were below 9%. Data from two participants were excluded due to missing values in three and five of the seven timepoints, respectively. Additionally, for five participants with a single missing value, we imputed the value using the average of the two adjacent timepoints. We log-transformed cortisol data prior to statistical analyses and visualization to correct for non-normal error distributions.

### 2.8 Statistical analyses

The analysis plan was registered on OSF (https://osf.io/w7b8x) after data collection but before data inspection. All analyses were conducted in R (v4.4.3; R Core Team, 2025). Effect sizes are reported as Cohen’s d, with positive values indicating effects in favor of the nature groups.

#### 2.8.1 Manipulation checks and retrospective ratings (H6)

To assess whether the stressor elicited clear stress responses we performed different manipulation checks (see Supplementary Methods and Results). To investigate participants’ perception of the environmental stimuli we tested whether perceived restorativeness and helpfulness in managing or recovering from a hypothetical stressful event differed by environment using a two-way ANOVA with the factors environment (nature vs. indoor) and time (pre vs. post-stress). We registered that nature environments would receive higher ratings than indoor environments (H6). In a separate, non-registered analysis, we examined whether participants retrospectively perceived the nature videos as more helpful for preparing for the stressor or recovering from the stressor. We used two independent t-tests to compare preparation ratings for groups 1 and 2 (pre-stress environment exposure) and recovery ratings for groups 3 and 4 (post-stress environment exposure).

#### 2.8.2 Exposure effects on overall stress response (H1)

Next, we investigated the registered hypothesis that nature (vs. indoor) exposure reduces overall stress (H1) using an area under the curve with respect to increase approach (AUCi; Pruessner et al., 2003). Essentially, the AUCi approach assumes that an individual has a baseline state of autonomic, hormonal and psychological arousal before exposure to a stressor and will: a) initially demonstrate an increase of arousal up to a specific peak (the reactivity phase); b) eventually returning to the original baseline, or some alternative homeostatic state (the recovery phase). The AUCi represents the total deviation from the original baseline, integrating both the magnitude and duration of the stress response. Separate AUCis were calculated for self-reported stress (STAI-SKD; t1-t4), HR (t1-t5), HRV (RMSSD; t1-t5) and salivary cortisol (t1-t7). We selected t4 and t5 as final time points of interest for self-reported and autonomic measures, as we expected recovery effects to emerge relatively shortly after the post-stressor phase (Ulrich et al., 1991). To assess group differences, we conducted two-way ANOVAs with the factors environment (nature vs. indoor) and time (pre vs. post-stress) as main effects. We predicted a less pronounced overall stress response in the nature compared to indoor groups (i.e., a main effect of environment).

#### 2.8.3 Exposure effects on stress reactivity (H2) and stress recovery (H3)

To test our registered hypotheses that nature (vs. indoor) exposure before the acute stressor attenuates stress reactivity (H2), and that nature (vs. indoor) exposure after the acute stressor enhances stress recovery (H3), we modeled individual trajectories over time for each repeatedly measured outcome (i.e., self-report, hormonal, and autonomic measures). We used conditional piecewise growth curve models (CPGCM; Grimm et al., 2016; Mirman, 2017) within a multilevel modeling framework (nlme package; Pinheiro, Bates & R Core Team, 2025), which allowed us to estimate separate reactivity and recovery slopes. We identified each participant’s peak stress response (i.e., knot-point) based on established procedures (Felt et al., 2017; Lopez-Duran et al., 2014). Using this peak, we split trajectories into a reactivity phase (from baseline to peak) and a recovery phase (from peak to final measurement). Reactivity and recovery were modeled using two linear time predictors. The reactivity predictor represented linear change from baseline to the individual peak, whereas the recovery predictor represented linear change from the peak to the final measurement. We tested whether these slopes differed by condition and registered that nature (vs. indoor) exposure would reduce stress reactivity (i.e., a less steep positive increase from baseline to peak) when presented before the stressor (H2), and enhance recovery (i.e., a steeper negative decrease from peak to final measure) when shown after (H3). These hypotheses were tested separately for self-reported (STAI-SKD), hormonal (cortisol), and autonomic (HR or HRV) indicators related to stress. We centered the models on the peak response and included baseline levels of the outcome variables as covariates. Fixed effects were specified for reactivity and recovery slopes, group (nature vs. indoor), and the relevant slope-by-group interaction (reactivity slope for H2; recovery slope for H3). Random effects were specified for intercepts, the reactivity and recovery slopes, and the interaction term (reactivity*group for H2, recovery*group for H3), to account for individual variation. H2 contrasted groups 1 (indoor-pre) vs. 2 (nature-pre), and H3 contrasted groups 3 (indoor-post) vs. 4 (nature-post) using dummy coding. Full model formulae, specifications and further details are reported in the Supplementary Methods and Results.

#### 2.8.4 Exposure effects on appraisal (H4) and rumination (H5)

Lastly, we tested our registered hypotheses that nature (vs. indoor) exposure before the acute stressor changes appraisal (H4), and that nature (vs. indoor) exposure after the acute stressor changes rumination (H5), using independent t-tests. For primary and secondary appraisal (PASA), we compared groups 1 (indoor-pre) and 2 (nature-pre), with the registered prediction that the nature group would report lower primary (e.g., threat) and higher secondary appraisal (e.g., competence). For positive and negative rumination (Thoughts Questionnaire), we compared groups 3 (indoor-post) and 4 (nature-post), with the registered expectations of lower negative and higher positive rumination in the nature group.

#### 2.8.5 Three-sided testing for H1-H6

In addition to null hypothesis significance testing (NHST), we applied three-sided testing (TST; Isager & Fitzgerald, 2025; Lakens, 2017). TST categorizes observed effects as meaningfully positive (superior), negative (inferior), practically equivalent to zero, or inconclusive. Whereas NHST only assesses whether an effect differs significantly from zero, TST evaluates whether it exceeds a predetermined smallest effect size of interest (SESOI) or falls within a SESOI-bounded range around zero that can be considered negligible in terms of its practical implications or scientific relevance. We liberally defined the SESOI as Cohen’s d = ± 0.2, corresponding to what is usually considered a small effect size. Although Cohen’s benchmarks have been criticized and should be interpreted with caution (Funder and Ozer, 2019), we adopted them as a pragmatic heuristic given the lack of established SESOI in acute stress and nature-stress research. Each effect is reported with its TST categorization as either “equivalent” (practically indistinguishable from zero, i.e., within the SESOI bounds), “inferior” (below the lower SESOI bound), “superior” (above the upper SESOI bound) or “inconclusive” (none of the above categories). For growth curve models, we based TSTs on differences in delta scores, as no established effect size measures exist for fixed effects in multilevel models. Full TST results are provided in the Supplementary Results.

## 3 Results

### 3.1 Manipulation checks and retrospective ratings (H6)

The acute stressor was retrospectively rated as stressful and elicited immediate stress responses across self-reported (STAI-SKD), hormonal (cortisol), and autonomic (HR and HRV) measures, with trajectories consistent with previous evidence (see Supplementary Results and Supplementary Fig. 1). Consistent with their distinct temporal dynamics, self-reported and autonomic indicators peaked during the anticipatory (t3a) and acute phase (t3b) of the MAST and showed significant reactivity and recovery slopes relative to their respective peaks in piecewise growth curve models. In contrast, cortisol responses exhibited the expected delayed trajectory, peaking approximately 20−25 min after stressor onset (t4), and likewise showed significant reactivity and recovery slopes relative to their peak response. Together, these patterns confirm the effectiveness of the stress manipulation across response systems while accounting for their different temporal dynamics. We additionally investigated participants’ perceptions of the virtual environments across several dimensions. Two-way ANOVAs revealed the main effect of environment to be significant for perceived restorativeness (F(1, 160) = 210.59, p < .001), helpfulness in managing (F(1, 160) = 226.88, p < .001), and helpfulness in recovering from a stressful event (F(1, 160) =197.24, p < .001). Consistent with our registration, nature (vs. indoor) videos were rated significantly higher on restorativeness (d = 2.28, 95% CI = [1.85, 2.82]), helpfulness in managing stress (d = 2.36, 95% CI = [1.86, 3.03]), and helpfulness in recovering from stress (d = 2.19, 95% CI = [1.76, 2.84]). All effects were of considerable magnitude and were significantly bounded above the upper SESOI (all p-values < 0.001), supporting their categorization as superior (see Supplementary Results and Supplementary Fig. 1). No significant main effect of time and no significant time*environment interactions (all p-values > 0.193) were observed in any of the analyses (see Supplementary Results). In other words, participants’ ratings of the environments did not differ by timing of video exposure across the measured dimensions and were consistently higher for nature than for indoor videos.

**Fig. 2:**
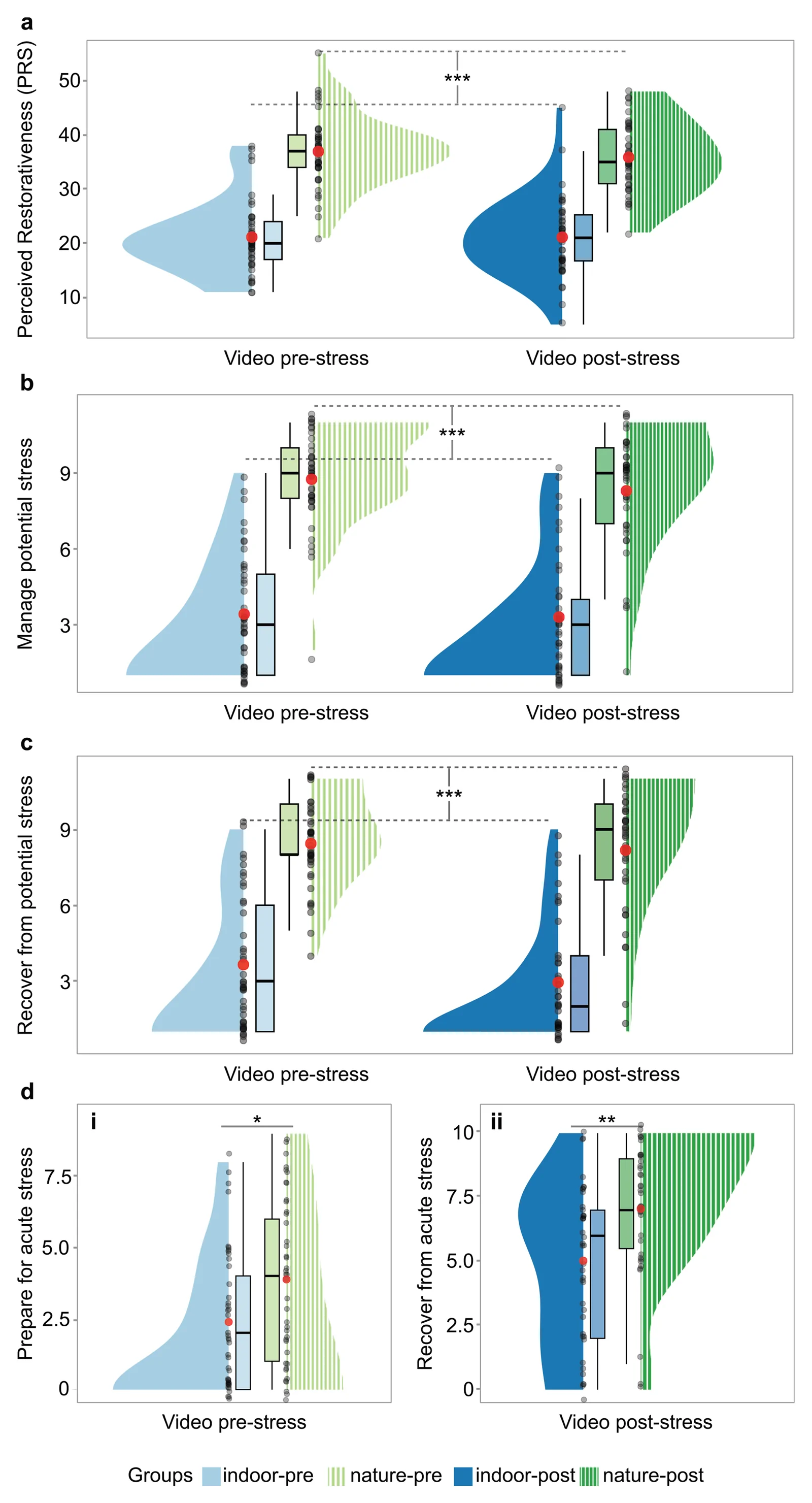
Group differences in retrospective ratings. Density plots depict individual responses (grey dots), and group means (red dots). Boxplots display the median, first and third quartiles, and whiskers extending to the 1.5x interquartile range. **(a)** Perceived Restorativeness Scale (PRS; sum score), with significantly higher ratings for the pooled nature vs. the pooled indoor groups. **(b)** Perceived helpfulness of the depicted environment for managing a hypothetical stressful scenario, with significantly higher ratings for the pooled nature vs. the pooled indoor groups. **(c)** Perceived helpfulness of the depicted environment for recovering from a hypothetical stressful scenario, with significantly higher ratings for the pooled nature vs. the pooled indoor groups. **(di)** Retrospective ratings of how much the videos before the acute stressor helped prepare for it with significantly higher ratings for the nature-pre vs. the indoor-pre groups. **(dii)** Retrospective ratings of how much the videos after the acute stressor helped to recover from it with significantly higher ratings for the nature-post vs. the indoor-post groups. *p < .05, **p < .01, ***p < .001. (For interpretation of the references to colour in this figure legend, the reader is referred to the Web version of this article.)

Additionally, we conducted exploratory t-tests comparing retrospective ratings of helpfulness in preparing for (indoor-pre vs. nature-pre) or recovering from (indoor-post vs. nature-post) the acute stressor. Nature videos received higher ratings than indoor videos for preparation, t(81) = 2.54, p = .013 (two-sided), d = 0.56, 95% CI = [0.14, 1.06] and recovery, t(78) = 3.22, p = .002 (two-sided), d = 0.72, 95% CI = [0.28, 1.23]. While the preparation effect was inconclusive in superiority testing (p = .11), the recovery effect significantly exceeded the upper SESOI (p = .026). Together, and consistent with our registered prediction, these findings indicate that nature videos were perceived as more restorative and were retrospectively rated as more helpful in preparing for and especially recovering from the acute stressor. Fig. 2 displays raincloud plots of ratings for (a) the perceived restorativness scale (PRS), (b) perceived helpfulness in managing, or (c) recovering from a hypothetical stressful event, as well as (d) retrospective ratings of how helpful the videos were for (i) preparing for or (ii) recovering from the acute stressor.

### 3.2 Exposure effects on overall stress responses (H1)

To test our registered hypotheses of lower overall stress responses in the nature vs. indoor groups (H1) we conducted two-way ANOVAs for each repeatedly measured response.

#### Self-reported responses

**Fig. 3:**
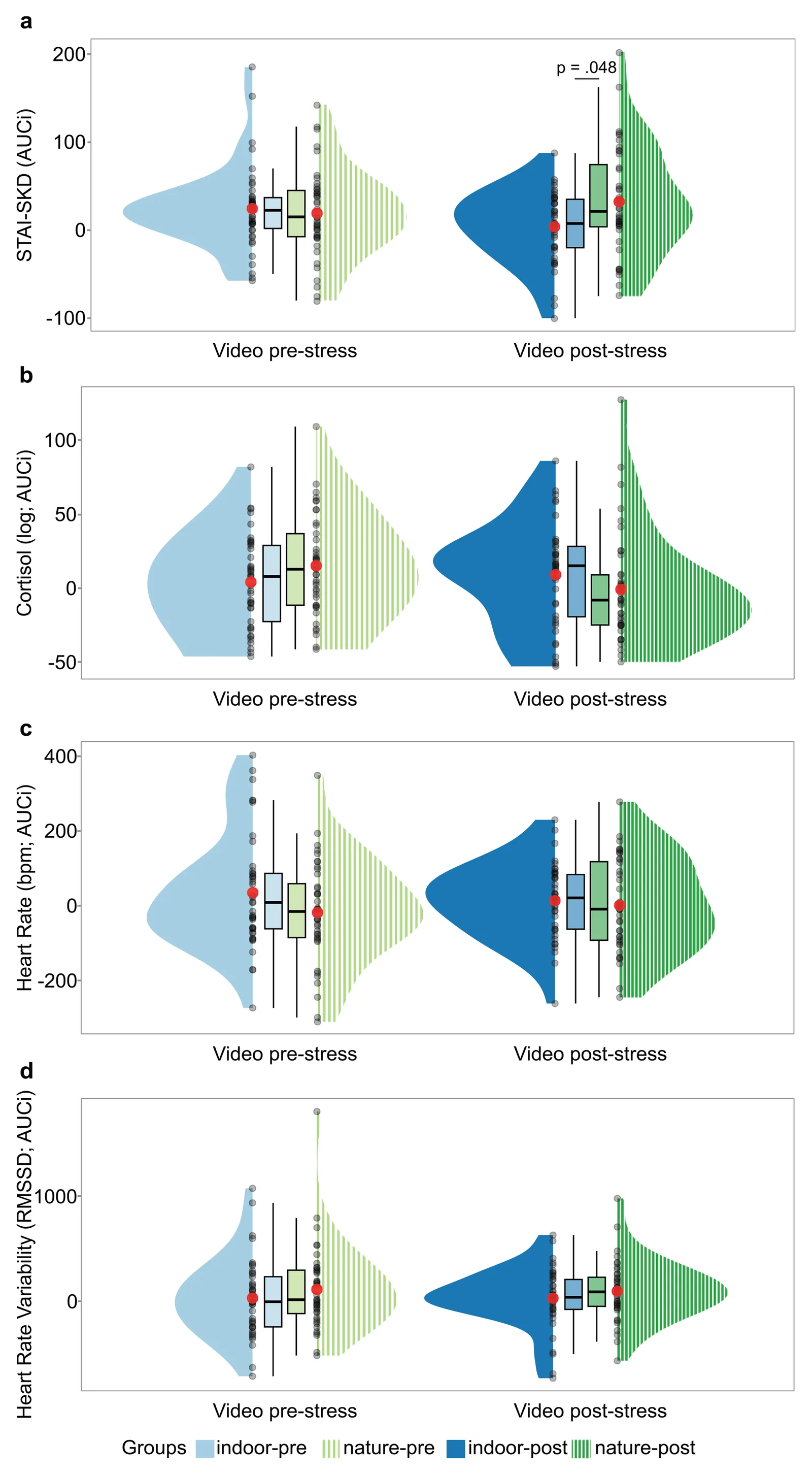
Group differences in overall stress responses measured as area under the curve with respect to increase (AUCi). Density plots depict individual values (grey dots), and group means (red dots). Boxplots display the median, first and third quartiles, and whiskers extending to the 1.5x interquartile range. **(a)** Self-reported responses (State Trait Anxiety Inventory, short form; STAI-SKD) **(b)** Hormonal responses (salivary cortisol, log-transformed) **(c)** Autonomic responses (heart rate, beats per minute; bpm) **(d)** Autonomic responses (heart rate variability, root mean square of successive differences; RMSSD). (For interpretation of the references to colour in this figure legend, the reader is referred to the Web version of this article.)

For the STAI-SKD, the two-way ANOVA revealed no main effects of environment or time (all p-values > 0.143; see Supplementary Results), but a significant environment*time interaction (F(1, 160) = 4.56, p = .034; Fig. 3a). To unpack this interaction, we conducted four pairwise comparisons: nature-pre vs. indoor-pre, nature-pre vs. indoor-post, nature-post vs. indoor-pre, and nature-post vs. indoor-post. After Bonferroni-Holm correction, only the nature-post vs. indoor-post comparison was significant, t(160) = −2.54, p = .048 (two-sided), d = −0.55, 95% CI = [−0.99, −0.12], indicating a higher overall self-reported stress response in the nature-post group.

#### Hormonal responses

For cortisol, the two-way ANOVA revealed no main effects of environment or time (all p-values > 0.305; see Supplementary Results), but a trend for the environment*time interaction (F(1, 158) = 3.80, p = .053). Repeating the same pairwise comparisons as for self-reported responses (two-sided, Bonferroni-Holm corrected) did not reveal any significant group differences.

#### Autonomic responses

For HR and HRV, the two-way ANOVAs revealed no significant main effects or interactions (all p-values > 0.106; see Supplementary Results).

We additionally conducted three-sided testing comparing the pooled nature and pooled indoor groups. Across all four stress outcomes, the results were inconclusive (see Supplementary Results and Supplementary Fig. 2), indicating that effects above or below our SESOI (d = ±0.2) could not be ruled out.

Thus, contrary to our registered hypotheses, we found inconclusive evidence for reduced overall stress responses in the nature compared to the indoor groups. Fig. 3 displays raincloud plots of overall stress responses (AUCi) for self-reported (STAI-SKD, a), hormonal (cortisol, b), and autonomic measures (HR, c; HRV, d) across groups.

### 3.3 Exposure effects on stress reactivity (H2) and stress recovery (H3)

We tested differences in stress reactivity (H2) and recovery (H3), using separate conditional piecewise growth curve models (CPGCM) for self-reported (STAI-SKD), hormonal (cortisol), and autonomic (HR and HRV) responses. Across all models, baseline scores significantly influenced peak responses (i.e., higher baselines were associated with higher peaks), reactivity slopes showed a clear change from baseline to peak, and recovery slopes indicated a significant change from peak to final measurement, confirming that the expected phases of stress reactivity and recovery were present (see Supplementary Results). To test our registered hypotheses that nature exposure reduces stress reactivity (H2) and facilitates recovery (H3), we examined the interactions of environment with the reactivity or recovery slopes for each repeatedly measured response.

#### Self-reported responses

**Fig. 4:**
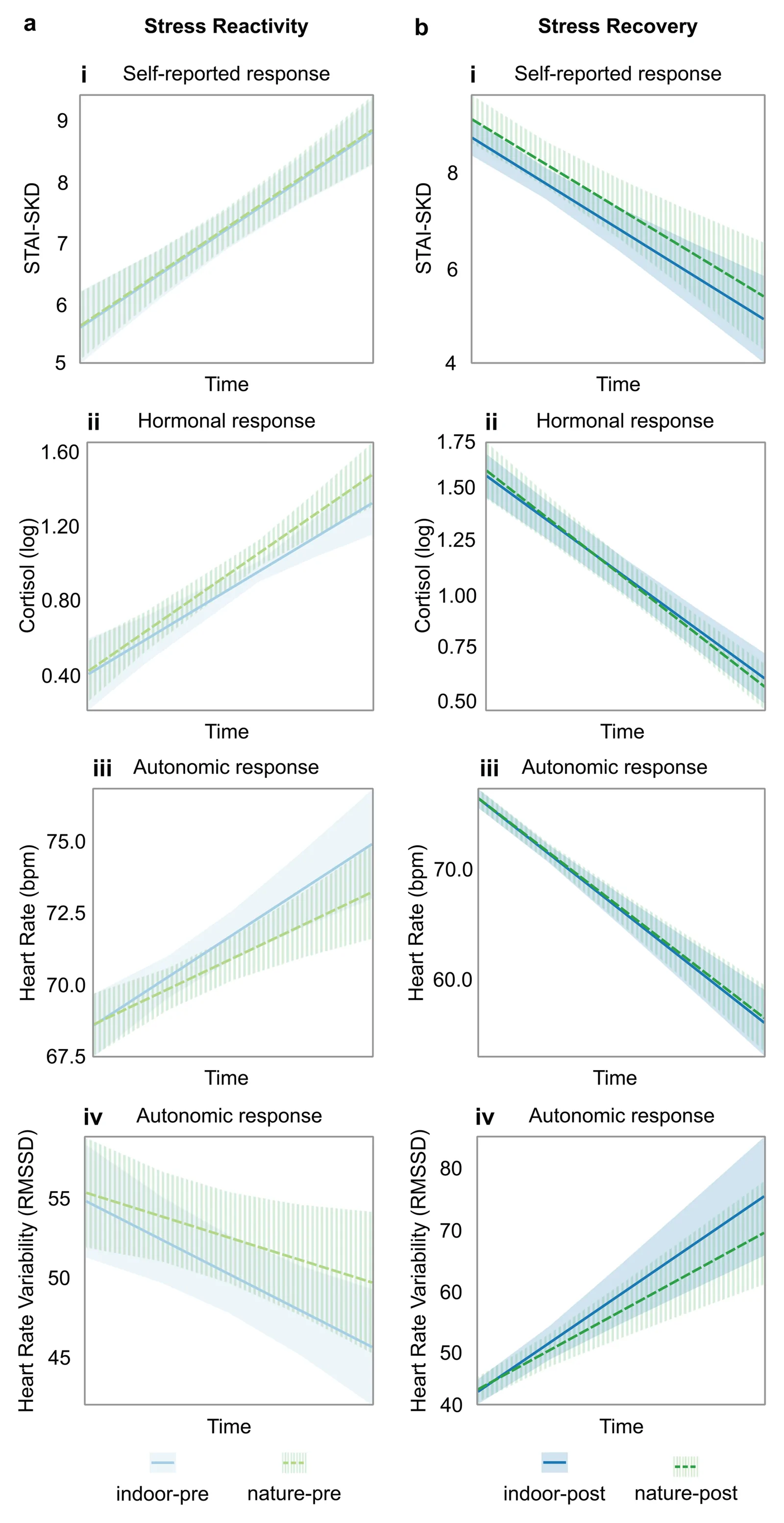
Group differences in stress reactivity and recovery. Stress responses are shown across self-reported measures (i: State Trait Anxiety Inventory, short form; STAI-SKD), hormonal responses (ii: salivary cortisol, log-transformed), and autonomic responses (iii: heart rate, beats per minute; bpm; iv: heart rate variability, root mean square of successive differences; RMSSD). Plots depict estimated slopes with 95% confidence intervals from conditional piecewise growth curve models. **(a)** Stress reactivity, comparing indoor-pre vs. nature-pre groups. Reactivity was modeled as the linear increase from baseline to peak response. **(b)** Stress recovery, comparing indoor-post vs. nature-post groups. Recovery was modeled as the linear decrease from peak response to the final measurement. (For interpretation of the references to colour in this figure legend, the reader is referred to the Web version of this article.)

Reactivity slopes for repeated STAI-SKD measures did not differ between indoor-pre and nature-pre groups (Fig. 4a(i); *b* = 0.003, SE = 0.20, t(329) = 0.014, p = .989, 95% CI = [−0.39, 0.40]). Similarly, recovery slopes for indoor-post vs. nature-post groups did not differ significantly (Fig. 4a(ii); *b* = 0.030, SE = 0.24, t(321) = 0.124, p = .901, 95% CI = [−0.45, 0.51]).

#### Hormonal responses

For cortisol, reactivity slopes did not differ between indoor-pre and nature-pre groups (Fig. 4b(i); *b* = 0.027, SE = 0.05, t(489) = 0.640, p = .522, 95% CI = [−0.06, 0.11]). Similarly, recovery slopes did not differ significantly for indoor-post vs. nature-post groups (Fig. 4b(ii); *b* = −0.016, SE = 0.02, t(477) = −0.717, p = .473, 95% CI = [−0.06, 0.03]).

#### Autonomic responses

Reactivity slopes did not differ between indoor-pre vs. nature-pre groups for HR (Fig. 4c(i); *b* = −0.569, SE = 0.46, t(321) = −1.248, p = .213, 95% CI = [−1.46, 0.33]), or HRV (Fig. 4c(ii); *b* = 1.169, SE = 1.29, t(321) = 0.901, p = .368, 95% CI = [−1.38, 3.72]). Similarly, recovery slopes for indoor-post vs. nature-post groups did not differ significantly for HR (4c(ii); *b* = 0.208, SE = 1.02, t(321) = 0.205, p = .838, 95% CI = [−1.79, 2.21]), or HRV (4d(ii); 2.17]). *b* = −2.073, SE = 2.16, t(321) = −0.961, p = .338, 95% CI = [−6.32,2.17]).

Three-sided tests on the peak-baseline difference for the indoor-pre and nature-pre groups, and on the peak-final measurement difference for indoor-post and nature-post groups were furthermore inconclusive (see Supplementary Fig. 3), suggesting that effect sizes above or below our SESOI (d = ±0.2) could not be ruled out. Thus, contrary to our registered hypotheses, we found inconclusive evidence for reduced stress reactivity or enhanced stress recovery in the nature groups across all outcomes. Fig. 4 displays estimated reactivity (a) and recovery (b) slopes from the CPGCM for the STAI-SKD (i), cortisol (ii), HR (iii), and HRV (iv) separated by indoor-pre vs. nature-pre (a) and indoor-post vs. nature-post (b) groups.

### 3.4 Exposure effects on appraisal (H4) and rumination (H5)

**Fig. 5:**
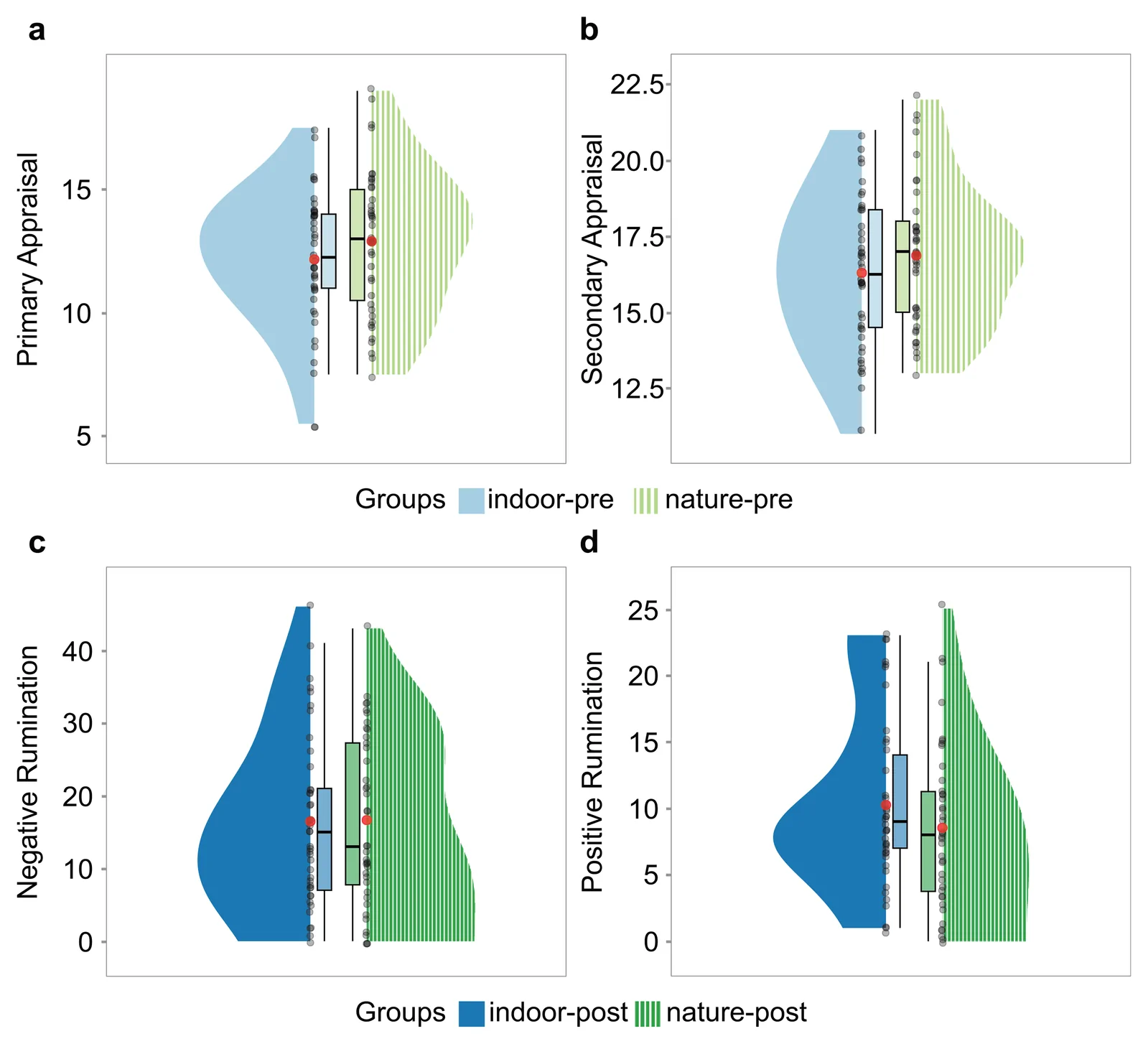
Group differences in appraisal and rumination related to the acute stressor. Density plots depict individual values (grey dots), and group means (red dots). Boxplots display the median, first and third quartiles, and whiskers extending to the 1.5x interquartile range. **(a)** Primary appraisal (perceived threat and challenge; sum score), comparing indoor-pre vs. nature-pre groups. **(b)** Secondary appraisal (perceived control and competence; sum score), comparing indoor-pre vs. nature-pre groups. **(c)** Negative rumination, comparing indoor-post vs. nature-post groups. **(d)** Positive rumination, comparing indoor-post vs. nature-post groups. (For interpretation of the references to colour in this figure legend, the reader is referred to the Web version of this article.)

To test differences in appraisal (H4) and rumination (H5) we ran independent sample t-tests for the nature and indoor groups. We observed no significant differences between the indoor-pre and nature-pre groups in primary appraisal (t(81) = −1.19, p = .881 (one-sided), d = −0.26, 95% CI = (−0.71, 0.19)) or secondary appraisal (t (81) = −1.08, p = .141 (one-sided), d = 0.24, 95% CI = [−0.68, 0.20)). Similarly, indoor-post and nature-post groups did not differ in negative rumination (t(79) = −0.07, p = .529 (one-sided), d = −0.02 95% CI = (−0.46, 0.44)) or positive rumination (t(79) = 1.23, p = .889 (one-sided), d= −0.27 95% CI = [−0.73, 0.19)). Exploratory two-sided tests likewise revealed no differences in the non-registered direction (see Supplementary Results). Three-sided testing was furthermore inconclusive (see Supplementary Fig. 4), indicating that effect sizes above or below our SESOI (d = ±0.2) could not be ruled out. Thus, contrary to our registered predictions, nature exposure had inconclusive effects on appraisal or rumination. Fig. 5 displays raincloud plots of (a) primary and (b) secondary appraisal ratings for the indoor-pre and nature-pre groups, as well as (c) negative and (d) positive rumination ratings for the indoor-post and nature-post groups.

## 4 Discussion

The present study investigated how virtual nature exposure affects stress reactivity and recovery, as well as associated emotion regulation strategies during acute stress. Using a randomized controlled design with a sizeable sample, determined based on a formal power analysis assuming a moderate effect size, participants retrospectively evaluated a virtual nature environment to appear to them as more restorative than an indoor control environment (large effect sizes). They also retrospectively reported that viewing nature was perceived as helping them prepare for and recover from an experimental stressor (moderate effect sizes). However, these retrospective impressions were not reflected in participants’ immediate physiological or self-reported stress responses, nor in their reported emotion regulation strategies. Contrary to our predictions, we observed no significant group differences in favor of nature in overall stress response, stress reactivity, stress recovery, appraisal or rumination. Yet, equivalence tests indicated that small (and sometimes moderate) effects could not be ruled out, leaving the null findings largely inconclusive.

### 4.1 Disconnect between retrospective and immediate effects

A key insight is the apparent disconnect between participants’ retrospective evaluations and their immediate physiological and psychological responses. Although the natural environment was consistently rated as highly restorative, these perceptions did not translate into significant differences in immediate stress responses. This suggests that brief, two-dimensional virtual nature exposure may exert no or subtle, context-dependent immediate effects. It is worth noting that this divergence, between the large differences in perceived restorativeness and the absence of effects on immediate stress markers, does not reflect a subjective-physiology mismatch, as immediate self-reported stress was also not significantly lower in the nature conditions. Rather, it appears instead to reflect a dissociation between immediate stress responses and retrospective environmental appraisal. The consistency of these findings across multiple stress indicators, combined with equivalence testing, indicates that any true effects are likely less prominent than previously reported or anticipated by theory. The observed effect sizes align with our recent meta-analysis on nature-induced analgesia (Steininger et al., 2026), which found significant but small-to-moderate effects of nature exposure on self-reported pain. Collectively, these findings underscore the need to critically reflect expectations regarding the potency of minimal or simulated nature interventions across different stress responses.

The discrepancy between retrospective ratings and immediate stress responses concurs with a broad literature showing that natural environments are almost invariably perceived as highly restorative, while effects on other outcomes are less consistent. For instance, a systematic review found that 95% of studies reported higher perceived restorativeness for nature compared with other environments (Browning et al., 2021). Notably, the review authors caution that such ratings may not reliably reflect actual health benefits and relying on perceived restorativeness as a primary outcome may overstate nature’s salutary effects. Retrospective ratings are also vulnerable to memory or recall biases (Coughlin, 1990) or expectancy effects shaped by cultural narratives that nature is restorative (Egner et al., 2020). Nevertheless, these ratings should not be dismissed, as they can provide insights into how individuals broadly experience and evaluate different environments. At the same time, the consistently higher perceived restorativeness of nature should be interpreted cautiously, particularly because it may not correspond to more immediate health outcomes, which tend to show smaller and more variable effects.

In line with this observation, higher perceived restorativeness and retrospective helpfulness ratings were not reflected in immediate stress outcomes. Across the sixteen comparisons assessing the overall immediate stress response, only one test reached statistical significance, unexpectedly suggesting higher self-reported stress in the nature-post compared to the indoor-post condition. However, this finding was not accompanied by similar effects in the remaining outcome measures, contrasted with the pattern observed in retrospective ratings, and was close to the conventional significance threshold (p = .048). Additionally, the corresponding three-sided test evaluating the overall effect of nature on self-reported stress remained inconclusive. Given the number of comparisons conducted and the lack of converging evidence across outcomes, this isolated finding should be interpreted with considerable caution, as a chance finding cannot be ruled out.

Consistent with this interpretation, we found no significant differences in stress reactivity or recovery between environments across stress markers. Additionally, retrospective ratings were not associated with alterations in immediate stress responses, indicating that participants’ evaluations of restorativeness and helpfulness did not correlate with actual physiological or self-reported changes (see Supplementary Results). The null finding for stress reactivity is novel, as no previous study has explicitly disentangled reactivity and recovery phases. It suggests that brief virtual nature exposure may provide little or no buffering of acute stress reactions when experienced prior to an aversive event. Unexpectedly, stress recovery was also unaffected, contrasting with earlier reports (e.g., Annerstedt et al., 2013; Yu et al., 2018) and the common proposed mechanism that nature facilitates stress recovery (Ulrich et al., 1991). However, the indication of potentially small effect sizes, together with recent inconclusive evidence, suggests that more nuanced interpretations are warranted.

### 4.2 Consistency and magnitude of virtual nature effects on stress

While several past studies report recovery effects across different stress outcomes after virtual nature exposure (Annerstedt et al., 2013; Brown et al., 2013; Laumann et al., 2003; S. H. Park et al., 2020; Schebella et al., 2019; Ulrich et al., 1991; Wang et al., 2016; Yu et al., 2018), more recent literature presents a less consistent picture. Since the planning phase and power analysis of the current study, several null or inconsistent findings (Jo et al., 2022; Knaust et al., 2022; Michels et al., 2022; Sun et al., 2023; Yin et al., 2022) and two systematic reviews have been published (Gentile et al., 2023; Spano et al., 2023), with conclusions varying by stress outcomes. For instance, virtual green environments versus non-green VR settings (Sun et al., 2023; Yin et al., 2022), and nature versus urban images (Michels et al., 2021) showed no effects on cortisol recovery. Similarly, contrasts between nature and urban VR (Jo et al., 2022; Mostajeran et al., 2021), natural versus non-natural room pictures (Michels et al., 2022), or a VR beach scenario versus passive control (Knaust et al., 2022) failed to show HR recovery benefits.

Two systematic reviews suggested that the most consistent effects of nature exposure emerge for self-reported stress and HRV (Gentile et al., 2023; Spano et al., 2023). Yet, even for these measures findings are mixed and depend on specific questionnaires or HRV indices. For instance, positive affect often improves after stress in virtual nature settings, whereas negative affect and anxiety show inconsistent results (Gentile et al., 2023), with half of the studies using the STAI reporting no group differences (Annerstedt et al., 2013; Li et al., 2020; Mostajeran et al., 2021; Yin et al., 2020). Similarly, HRV findings are favorable in some studies (Annerstedt et al., 2013; Brown et al., 2013; Liszio et al., 2018; Yin et al., 2020, 2022; Yu et al., 2018) but remain heterogeneous due to variations in HRV metrics and the presence of null or negative results (Alvarsson et al., 2010; Anderson et al., 2017; Michels et al., 2022; Snell et al., 2019). Overall, the literature shows substantial variability. In addition to differences in study design, including the choice of study populations or the types of acute stressors and nature stimuli employed, this variability may partly reflect limited consideration of expected effect sizes. This does not imply that the effects are absent but suggests that they may be subtle and context-dependent, and thus sometimes remain undetected.

Consistent with this assumption, our equivalence tests could not rule out small, and sometimes medium, effect sizes. Although previous systematic reviews have effectively characterized the evidence landscape (Gentile et al., 2023; Spano et al., 2023), the field would benefit from meta-analytic syntheses (Schmidt, 1992) to clarify expected effect sizes across outcomes and methodological choices, and address a key practical question: if the effects of virtual nature on acute stress are small, are they meaningful in practice? Defining a smallest effect size of interest is challenging, and our own equivalence threshold relied on benchmarks with acknowledged limitations (Funder and Ozer, 2019). For self-reported stress in the current study, the chosen benchmark of a small effect (d = 0.2) would translate to a difference of approximately 0.5 points between the nature and indoor groups on the change from baseline to stress peak. Given that participants, on average, showed an approximate 2.5-point increase from baseline to stress peak, such an effect would entail a relatively small difference in stress reactivity between groups. To our knowledge there is no established consensus in stress research regarding whether a difference of this magnitude is practically meaningful, highlighting the need for empirically and contextually informed benchmarks in future research. These benchmarks could be established through empirically grounded approaches, such as anchor-based methods (Anvari & Lakens, 2021), or through expert consensus. For example, in clinical pain research, consensus statements have been developed to aid the interpretation of what constitutes a meaningful change in self-reported pain (Dworkin et al., 2008). Importantly, these benchmarks may vary depending on the populations under investigation (White et al., 2023). Small effects meaningful at the population level (that potentially impacts millions of people) may be insufficient for at-risk groups or patients with existing physical or mental health conditions. At the same time the inconsistent study landscape raises important questions about the contextual factors that determine when nature may be effective.

### 4.3 Contextual factors influencing observed effects

From a methodological perspective, the choice of nature stimulus may be critical. Two-dimensional virtual nature might be insufficient, and in-situ contact (i.e., direct, physical presence in a real natural environment) or immersive virtual reality (VR) might be required. Indeed, several studies link in-situ exposure to changes in stress-related markers (Beil & Hanes, 2013; Hunter et al., 2019; Lee et al., 2014; B.-J. Park et al., 2007; Sudimac et al., 2022; Van Den Berg & Custers, 2011). Moreover, compared to 2D nature, VR enhances enjoyment, immersion, and presence (Newman et al., 2022; Yeo et al., 2020). Importantly, VR systems are often characterized by sensorimotor contingencies enabled through head or body movement tracking, which mimic perceptual experiences encountered in real-world environments (e.g., moving one’s head changes direction of gaze in a typical VR system, but not in a 2D presentation, Slater, 2009). In addition, they can differ from conventional 2D presentations in several objective properties that increase sensory fidelity, including a wider field of view, stereoscopic depth cues, and spatialized auditory inputs (Bowman & McMahan, 2007). These features are closely linked to users’ experiences of immersion and presence (Slater, 2009). Presence is commonly described as the subjective feeling of “being there” in a virtual environment (Sanchez-Vives & Slater, 2005) and may play an important role in the effectiveness of virtual nature. It is conceptually related to the notion of “being away”, which is considered a central component of the restorative potential of natural environments (Kaplan, 1995), as it enables individuals to mentally disengage from stressful and taxing demands. This experience may be further enhanced by providing richer sensory input (e.g., haptic feedback, olfactory stimulation, etc.), which was not considered in the present study.

Indeed, in the context of pain, which shares physiological and self-reported responses with stress, our recent meta-analysis found indications that higher stimulus immersiveness (operationalized as the number of engaged sensory modalities) is linked to greater effectiveness in pain reduction (Steininger et al., 2026). However, studies comparing presentation modes in acute stress research remain limited and inconclusive. While some studies report benefits for immersive nature (Liszio et al., 2018), others find inconsistent or absent differences (Ascone et al., 2025; Knaust et al., 2022; Mostajeran et al., 2021). Similarly, in-situ studies remain mixed and appear to depend on the specific ways individuals interact with nature (Mygind et al., 2021).

Beyond the nature stimulus, the use of a virtual indoor control condition may have obscured differences previously reported when comparing nature with urban environments. While many past studies have compared nature to urban settings (Chan et al., 2021; Hedblom et al., 2019; Mostajeran et al., 2021; Schebella et al., 2019; Spano et al., 2023; Sun et al., 2023), we deliberately avoided urban controls because their often inherently stressful characteristics could confound interpretation. Importantly, prior studies using indoor control conditions have still found beneficial effects of nature, including changes in negative affect (Anderson et al., 2017), HRV (Annerstedt et al., 2013), and cortisol (Yin et al., 2022). Moreover, a recent neuroimaging study from our lab using the same nature and indoor stimuli found stronger effects of nature on neural and self-reported indicators of pain processing (Steininger et al., 2025). Consistent with these findings, participants in the current study rated nature as considerably more restorative and helpful for coping with the stressor. Collectively, this suggests that using a virtual indoor scenario constitutes an appropriate control and unlikely explains our null findings.

### 4.4 Effects of virtual nature on stressor-specific emotion regulation

Beyond the absence of significant effects of virtual nature on self-reported, hormonal, and autonomic stress measures, we observed no significant impacts on appraisal and rumination. To our knowledge, no previous study has assessed stressor-specific response- (appraisal) and antecedent-focused (rumination) emotion regulation strategies. Thus, our observation that virtual nature exerts small or negligible impacts on these processes represents a novel contribution. Interestingly, it contrasts with evidence that nature exposure can reduce ruminative tendencies (Bratman et al., 2015, 2021; Lopes et al., 2020). However, these studies investigated in-situ nature exposure and general, undirected rumination, which may differ from the stressor-specific ruminative thoughts measured here.

### 4.5 Study limitations

Despite several methodological and conceptual strengths, this study also has limitations. First, we used a two-dimensional virtual nature stimulus, and it is possible that a more immersive VR nature scenario or additional sensory stimulation (e.g., olfactory or tactile cues) might produce stronger effects. Second, although the sample was sizeable, the study was powered for medium effects and may have been underpowered for the potentially smaller true effects in the context of virtual nature and acute stress. A within-participant design could have increased statistical power, but repeating the same stress task for the same participant introduces other challenges (e.g. habituation effects). Third, the effectiveness of virtual nature exposure may depend on participants’ preferences for, or prior experiences with, different environmental settings. Although using the same nature stimulus for all participants enhanced standardization, we recognize that the specific context may be more appealing to some individuals than others based on variations in their lived experiences, preferences, or familiarity with specific environments. For instance, Yin et al. (2022) found that stress recovery effects were present for virtual desert but not virtual green environments among participants who grew up in desert regions. Although our nature stimulus resembled environments commonly encountered in the country of investigation, variability in participants’ prior experiences across groups may have reduced the observed effects. In line with this, a further limitation is that we did not directly assess such preferences for, or prior experiences with, different environmental settings. However, as reported in the Supplementary Information, we found no evidence that related constructs such as nature connectedness and lifetime urban exposure moderated the effect of environment (nature vs. indoor) on immediate stress-related outcomes. This suggests that the observed null effects of environmental condition are unlikely to be explained by systematic differences in nature connectedness or prior lifetime urban exposure. Notably, although these moderation effects were not significant, exploratory analyses indicated that higher levels of lifetime urban exposure were associated with stronger cortisol responses to the acute stressor across study groups, suggesting that prior exposure to urban environments may be related to heightened hormonal stress responses in general. Lastly, our smallest effect size of interest relied on conventional effect-size benchmarks (Cohen, 1988), which have been subject to justified critique (Funder and Ozer, 2019). The field should move towards more empirically informed thresholds and critically discuss what constitutes a practically meaningful effect.

### 4.6 Conclusion and implications

Overall, our study provides inconclusive evidence regarding the potential of virtual nature to influence stress reactivity, stress recovery, and both antecedent- (appraisal) and response-focused (rumination) emotion regulation strategies during acute laboratory stress. Exposure to virtual nature versus indoor scenes, before or after the stressor, did not produce significant changes in these measures. However, equivalence tests indicate that small (to medium) effects cannot be ruled out. Given the notable variability and inconsistency in prior studies, such small effects may be characteristic of the nature-stress relationship under controlled laboratory conditions. Future research should critically investigate factors contributing to this variability, including interindividual differences in responsiveness to nature, stimuli tailored to personal preferences or lived experiences, and potentially longer, repeated and more immersive exposures. If virtual nature generally yields small effects on acute stress, it is critical to determine under which conditions such effects become practically meaningful and whether they can be reliably observed in future studies with larger samples and well-controlled designs. Considering that virtual nature exposure is an easily implementable intervention with minimal side effects, its potential in ameliorating stress warrants further investigation, particularly for individuals that can’t visit nature in-situ. Even small effects could have a relevant impact at the population level benefiting large numbers of people.

## Supplementary Material

**Appendix A. Supplementary data**

Supplementary data to this article can be found online at https://doi.org/10.1016/j.jenvp.2026.103181.

Supplementary Information

## Data Availability

Single-time-point self-report data (appraisal, rumination, restorativeness, retrospective ratings, trait measures), repeatedly assessed self-reported state anxiety (STAI-SKD; t1-t6), salivary cortisol concentrations (t1-t7), and averaged heart rate and heart rate variability indices for each time-segment (t1-t5) extracted from the preprocessed ECG time-series, as well as individual knot-points used for conditional piecewise growth curve modeling (CPGCM), are available at https://osf.io/tja8u.

## Code availability

The code for the main and supplementary analyses is available at https://osf.io/tja8u.
