# Supplementary material for: Brief virtual nature exposure and acute stress: inconclusive evidence regarding stress reactivity, recovery, and emotion regulation
Source: J Environ Psychol. Author manuscript; Available in PMC 2026 Sep 1. (PMC7619404; doi:10.1016/j.jenvp.2026.103181)
Supplement: Supplementary file 1 [file EMS217800-supplement-Supplementary_Information.docx]

**Supplementary Information for**

Brief virtual nature exposure and acute stress: inconclusive evidence regarding stress reactivity, recovery, and emotion regulation

Steininger, M.O.^1^, Nitschke, J.P.^1^, Schenk, L.^1^, White, M.P. ^2,3,4,5^, Lamm, C.^1,3,5*^

^1^Department of Cognition, Emotion, and Methods in Psychology, Faculty of Psychology, University of Vienna, Vienna, Austria

^2^ Department of Clinical & Health Psychology, Faculty of Psychology, University of Vienna, Vienna, Austria

^3^ Cognitive Science Hub, University of Vienna, Vienna, Austria

^4^ European Centre for Environment and Human Health, University of Exeter, UK

^5^ Environment and Climate Research Hub, University of Vienna, Vienna, Austria

* Corresponding author: Claus Lamm

**Email:** claus.lamm@univie.ac.at

**Supplementary Methods**

*Power analysis*

The sample size was determined based on prior research comparing nature interventions to control conditions on acute stress responses, measured by changes in cortisol (Annerstedt et al., 2013; Liszio et al., 2018; Thoma et al., 2013). We performed a-priori power estimations using G*Power (Faul et al., 2009) with a type-I error probability of α = .05, a power of 1-beta = .8, and a moderate effect size of f = 0.27, for a four-group one-way ANOVA, resulting in a required sample size of N = 156. Anticipating exclusions and dropouts, we recruited 10 additional participants, yielding a final sample of N = 167. Although we did not conduct separate power analyses for the remaining dependent variables (i.e., self-reported and autonomic measures), prior meta-analyses suggested that psychological interventions (e.g., music or mindfulness) elicit stronger and more consistent effects on self-reported and autonomic indicators of stress compared to hormonal markers . Therefore, we based our power calculation on the most conservative outcome (i.e., cortisol).

*Participants*

Participants were recruited through online and offline advertisements, as well as a participant database at the University of Vienna (<https://spp.cogsci.univie.ac.at/>), and received a compensation of 20 to 30€ for taking part in the experiment depending on the individual duration of the session. Exclusion criteria included any current or past psychiatric or neurological disorder, severe or chronic illness, conditions affecting pain perception, or chronic pain. Participants were also excluded if they were regular smokers, regularly consumed more than 10 standard units of alcohol per week, or had used recreational drugs within the past three months. Female participants were ineligible if they were pregnant or using hormonal contraception. Additionally, participants were excluded if they regularly took prescription medication, were enrolled in pharmacological studies, were psychology students beyond their second semester, or reported moderate to poor German language skills.

*Virtual stimuli*

Virtual stimuli were created by a proficient graphics designer. We quantified visual properties using a deep-learning based semantic segmentation approach (Aikoh et al., 2023) to automatically estimate the Green View Index (GVI; Aoki, 1991). Semantic segmentation categorizes each pixel in an image with a predefined class label (e.g., tree, grass, desk). We performed semantic segmentation in Python (PyTorch 2.10.0, transformers 5.6.2) using a pretrained SegFormer-B0 model (Xie et al., 2021) trained on the ADE20K dataset (Zhou et al., 2017), which comprises 150 categories. Automatic segmentation has been shown to correlate highly (r > 0.96) with conventional manual annotation methods (Aikoh et al., 2023). The GVI was calculated as the proportion of total image pixels classified as vegetation using four categories (“tree”, “grass”, “plant”, “flower”). We additionally estimated the proportion of pixels corresponding to bluespace (categories: “water”, “sea”, “river”, “waterfall”, “lake”) and visible sky (category: “sky”). We extracted and analyzed a single representative frame from each video (nature and indoor), as camera position and scene composition remained constant throughout, making a single frame representative of the full video. As intended during stimulus construction, the nature video contained a high proportion of nature-related categories with a GVI of 0.372, bluespace proportion of 0.183, and sky proportion of 0.438. In contrast, the indoor video contained no pixels classified as vegetation, bluespace, or sky (all values = 0.000).

We further created soundscapes that matched the visual content of the videos and included auditory elements typical of outdoor environments in the country of investigation (e.g., calls of native species) and office-based indoor settings. We analyzed auditory properties across the full duration of each soundscape in Python (librosa 0.11.0; scikit-maad 1.5.2). We computed peak amplitude, root-mean-square level (RMS; frame-wise mean RMS), dynamic range (DR), and crest factor (CF). Peak amplitude indicates the highest signal level, while RMS assesses the average signal level. DR quantifies the difference between louder and quieter proportions of the signal and was defined as 20 log_10_(P_95_/P_5_; where P_95_ and P_5_ denote the 95^th^ and 5^th^ percentiles of the absolute amplitude distribution). CF was defined as the ratio of peak to RMS amplitude. The nature soundscape exhibited a peak level of -6.46 dBFS, an RMS level of -34.34 dBFS, a DR of 36.22 dB, and a CF of 27.88 dB. The indoor soundscape showed a peak amplitude of -10.31 dBFS, an RMS level of -32.61 dBFS, a DR of 35.75 dB, and a CF of 22.30 dB. We furthermore assessed the spectral centroid as an index of acoustic brightness, which was higher in the nature soundscape (5008 Hz) than in the indoor soundscape (2793 Hz). Lastly, we assessed the acoustic diversity index (ADI; Villanueva-Rivera et al., 2011), which quantifies the complexity and richness of a soundscape, with higher values indicating greater biological acoustic diversity. The ADI was 1.67 for the nature and 0.09 for the indoor soundscape. Thus, the nature soundscape was characterized by a more dynamic, spectrally brighter, and acoustically diverse environment compared to the indoor condition, which exhibited more constant, lower-frequency, and acoustically uniform stimulation.

*Self-reported measures*

We repeatedly assessed self-reported stress using the German short form of the State-Trait Anxiety Inventory (STAI-SKD; Englert et al., 2011). The STAI-SKD includes five items asking participants to rate on a 4-point scale (1 = “not at all” to 4 = “very”), how much each statement applies to their current emotional state (e.g., “I am tense”). Participants completed the questionnaire at seven time points relative to stressor onset: after baseline ECG (t1, -10 minutes), after the first video intervention (t2, 0 minutes), after the Maastricht Acute Stress Task (MAST) preparation phase (t3a, 5 minutes), after MAST acute stress phase (t3b, 15 minutes), after the second video intervention (t4, 25 minutes), after stress-related questionnaires (t5, 35 minutes), and after the prosocial effort task (t6, 55 minutes). We operationalized state anxiety as the sum of all five items for each time point, with Cronbach’s α ranging from .69 to .84 across timepoints.

We measured primary and secondary appraisal of the acute stress phase using the Primary and Secondary Appraisal Scale (PASA; Gaab et al., 2005), administered immediately after the MAST preparation phase (t3a), when participants were informed about the upcoming stressor. The PASA includes four subscales: two measuring primary appraisal — “Threat” (e.g., “I do not feel threatened by the situation”) and “Challenge” (e.g., “I don’t care about this situation”) — and two measuring secondary appraisal — “Self-Concept of Own Competence” (e.g., “In this situation I know what I can do”) and “Control Expectancy” (e.g., “It mainly depends on me whether the expert judges me positively”) — each comprising 4 items. Items 1, 6, 7, 9 and 10 were reverse-coded due to inverse wording. We computed sum scores for each subscale and averaged “Threat” and “Challenge” to represent primary appraisal (Cronbach’s α = .77), and “Self-Concept of Own Competence” and “Control Expectancy” to represent secondary appraisal (Cronbach’s α = .71). Participants rated each item on a 6-point scale (1 = “completely wrong” to 6 = “completely right”).

We assessed post-event rumination after the second video intervention (t5) using an adapted version of the Thoughts Questionnaire (Edwards et al., 2003). Participants rated how often they had positive (e.g., “How well I handled the situation”) and negative (e.g., “How many mistakes I made”) thoughts about the acute stress phase since its conclusion, using a 5-point scale (0 = “never” to 4 = “very often”). The statements related directly to the acute stressful situation. We modified the wording of four statements (1, 5, 10 and 20 out of 27) to better align with the context of the MAST. We operationalized positive (11 items, Cronbach’s α = .82) and negative rumination (16 items, Cronbach’s α = .89) by calculating sum scores for each subscale.

We measured perceived restorativeness at the end of the experiment (t7) using the short form of the Perceived Restorativeness Scale (PRS; Berto, 2005; Hartig et al., 1997), after participants re-watched 1.5-minute versions of the virtual environments. The PRS includes five items, each reflecting one of the core dimensions proposed by Attention Restoration Theory (ART; Kaplan, 1995): “being-away”, “fascination”, “coherence”, “scope” and “compatibility”. Participants rated each item (e.g., “This is a place which is far away from everyday demands and where I would be able to relax and think about what interests me”) on an 11-point scale (1 = ”not at all” to 11 = “very much”). We calculated a sum score across all five items to operationalize perceived restorativeness (Cronbach’s α = .65). In addition, participants retrospectively rated how stressful they experienced the MAST (0 = “not at all” to 10 = “very stressful”), how much they believed whether being in the respective environment would help them to manage (single item) or recover (single item) from a hypothetical stressful event (0 = “not at all” to 10 = “very much”) and how much viewing the virtual environments helped them prepare for (single item; groups 1 and 2) or recover from (single item; groups 3 and 4) the acute stressor (0 = “did not help at all” to 10 = “helped very much”).

Furthermore, we conducted exploratory analyses to examine whether participants’ levels of nature connectedness and lifetime urban exposure predicted immediate responses to stress. Nature connectedness was assessed using the Nature Connection Index (NCI; Richardson et al., 2019). The NCI comprises six items (e.g., “Being in nature makes me very happy”, Cronbach’s α = .83 in the current sample), rated on a 7-point scale ranging from 1 = “completely disagree” to 7 = “completely agree”. Following previous recommendations (Richardson et al., 2019), we estimated an overall index of nature connectedness using a weighted scoring approach, with higher scores indicating greater nature connectedness. In line with previous research (Lederbogen et al., 2011), lifetime urban exposure was assessed by asking participants to report the number of years they had lived in cities (>100.000 inhabitants), towns (10.000-100.000 inhabitants), or rural areas (<10.000 inhabitants), summing up to their current age. Individual scores were calculated by weighting the reported years in each area (cities * 3, towns * 2, rural areas * 1) and dividing the sum of these weighted years by participants’ current age. This resulted in an index ranging from 1 to 3, where lower and higher values indicate predominantly rural or urban lifetime exposure, respectively.

*Piecewise growth curve models*

To model stress reactivity and recovery, we conducted conditional piecewise growth curve models (CPGCMs) within a multilevel modeling framework (Grimm et al., 2016; Mirman, 2017) using the nlme package (Pinheiro et al., 2025) in R (v4.4.3; R Core Team 2025). We applied CPGCM with landmark registration, which accounts for individual differences in the timing of peak responses (Lopez-Duran et al., 2014). For each participant and outcome, we identified a stress peak (knot-point) that defined the transition between reactivity (baseline to peak) and recovery (peak to last measurement). We then created two time variables representing the linear slope from baseline to peak (reactivity), and the linear slope from peak to last measurement (recovery). We determined knot-points by visually inspecting participant-specific trajectories for each outcome measure. If no clear peak was observable, we used the peak of the average trajectory across participants for the respective outcome. To facilitate interpretation, we centered the models on the peaks so that intercepts represented the peak stress response. Sensitivity analyses using the average peak across all participants (instead of landmark registration) yielded results consistent with the landmark-based approach.

For the manipulation check, we assessed whether the stressor elicited a clear reactivity and recovery response. To this end, we included baseline levels, reactivity slopes, and recovery slopes as fixed effects, and specified random effects for intercepts, reactivity slopes, and recovery slopes to account for individual variation (Supplementary Table 1 for model formulae). For Hypotheses 2 and 3, we expanded the basic model by adding condition (dummy-coded: group 1 vs. 2 for H2; group 3 vs. 4 for H3) and the respective interaction term (i.e., reactivity*condition for H2; recovery*condition for H3) as fixed effects, with corresponding random effects. Models for H2 included participants who viewed the videos before the stressor (groups 1 vs. 2), while models for H3 included participants who viewed the videos after the stressor (Supplementary Table 4 and 5 for model formulae).

**Results**

*Manipulation check*

To investigate whether the stressor elicited self-reported and physiological responses, we conducted several analyses. First, we tested participants’ retrospective ratings of the task’s stressfulness (M = 6.43, SD = 2.44) against zero. A one-sample t-test revealed a significant effect, t(163) = 33.73, p < .001, indicating that the participants perceived the stressor as stressful. Second, we modeled physiological and self-reported stress responses using piecewise growth curve models (Supplementary Methods and Supplementary Table 1). We estimated separate models for each outcome (STAI-SKD, cortisol, HR, HRV). Confirming the stressors’ effectiveness, we found significant linear reactivity slopes for STAI-SKD (*b* = 1.03, SE = 0.09, t_(654)_ = 11.798, p < 0.001, 95% CI = [0.87 , 1.21]), cortisol (*b* = 0.18, SE = 0.02, t_(970)_ = 9.813, p < 0.001, 95% CI = [0.15, 0.22]), HR (*b* = 1.86, SE = 0.22, t_(646)_ = 8.509, p < 0.001, 95% CI = [1.44, 2.30]), or HRV (*b* = -2.44, SE = 0.42, t_(646)_ = -5.819, p < 0.001, 95% CI = [-3.27, -1.62]). Similarly, we found significant linear recovery slopes for STAI-SKD (*b* = -1.29, SE = 0.10, t_(654)_ = -12.899, p < 0.001, 95% CI = [-1.49 , -1.09]), cortisol (*b* = -0.23, SE = 0.01, t_(970)_ = -21.726, p < 0.001, 95% CI = [-0.25, -0.21]), HR (*b* = -10.20, SE = 0.52, t_(646)_ = -19.539, p < 0.001, 95% CI = [-11.23, -9.17]), or HRV (*b* = 9.07, SE = 0.69, t_(646)_ = 13.033, p < 0.001, 95% CI = [7.71 , 10.44]). The results indicate significant stress reactivity (increases in STAI-SKD, cortisol, HR; decreases in HRV) and subsequent recovery (decreases in STAI-SKD, cortisol, HR; increases in HRV) across all measures. This interpretation is further substantiated by inspecting the trajectory plots of all dependent outcomes in Supplementary Figure 1.

**Supplementary Figure 1**. Trajectories for self-reported (a, STAI-SKD), hormonal (b, cortisol), and autonomic (c, heart rate; d, heart rate variability) stress measures across timepoints (t; numbers in brackets indicate minutes relative to stressor onset). The grey shaded area indicates the acute stress task (MAST), including the anticipation phase (t3a) and acute stress phase (t3b). Error bars represent standard errors of the mean at each measurement. bpm = beats per minute; RMSSD = root mean square of successive differences

As shown in the figure, self-reported stress (panel a) peaked at the end of the anticipation phase (t3a) and slightly decreased after the stressor was terminated (t3b), potentially reflecting relief after completing the task (ratings were obtained at the end of each phase). Heart rate (panel c) was highest during the active stress phase (t3b), whereas heart rate variability (panel d) reached its lowest levels (i.e., highest stress) during the anticipation phase and the acute stress phase (t3b), followed by a marked drop after stressor termination. Cortisol (panel b) exhibited a lagged pattern, with a clear peak 20-25 minutes after stress onset (t4), consistent with established findings (Kirschbaum et al., 1995). Notably, cortisol, heart rate and subjective stress operate on different temporal scales. While self-reported stress, HR and HRV changes occur rapidly, cortisol responses develop more slowly, peaking around 20-25 minutes after stress onset. Our CPGCM accounts for these temporal differences by allowing outcome-specific peak timing. In addition, the employed landmark registration approach accounts for interindividual differences in peak responses within each measure. In line with our registration, we therefore used outcome-specific time windows to model reactivity, recovery, and overall stress across measures.

Additionally, we conducted three-sided testing (TST) for all reactivity and recovery effects. To this end, we computed difference scores between baseline and peak (Δreactivity) and between peak and last measurement (Δrecovery). We used difference scores rather than fixed-effects estimates from the CPGCMs for TST, as there is no consensus regarding standardized effect size metrics in mixed-effects modeling. As a manipulation check and sensitivity analysis, we conducted one-sample t-tests on these difference scores across all participants to examine whether significant reactivity and recovery effects were present (Supplementary Table 2 for descriptive statistics). All outcomes showed significant deviations from zero: STAI-SKD (Δreactivity: t(163) = 14.72, p < .001; Δrecovery: t(163) = 15.81, p < .001), cortisol (Δreactivity: t(161) = 9.7, p < .001; Δrecovery: t(161) = 26.1, p < .001), HR (Δreactivity: t(161) = 9.56, p < .001; Δrecovery: t(161) = 23.68, p < .001), and HRV (Δreactivity: t(161) = -6.81, p < .001, Δrecovery t(161) = -15.20, p < .001). Together, these findings provide converging evidence that the stressor elicited robust self-reported, hormonal, and autonomic stress responses.

**Supplementary Table 1**

*Linear mixed model results from piecewise growth curve models of stress reactivity and recovery across the full sample. Outcomes include self-reported (STAI-SKD), hormonal (cortisol), and autonomic (heart rate and heart rate variability) measures.*

|  | **STAI-SKD** | | | | | **Cortisol (log)** | | | | | **Heart rate (BPM)** | | | | | **Heart rate variability (RMSSD)** | | | | |
| --- | --- | --- | --- | --- | --- | --- | --- | --- | --- | --- | --- | --- | --- | --- | --- | --- | --- | --- | --- | --- |
| **Fixed Effects** | est | SE | CI | t | p | est | SE | CI | t | p | est | SE | CI | t | p | est | SE | CI | t | p |
| Intercept | 3.39 | 0.39 | 2.61  4.17 | 8.55 | <.001 | 0.59 | 0.06 | 0.47  0.72 | 9.60 | <.001 | 6.31 | 1.34 | 3.68  8.94 | 4.7 | <.001 | -0.45 | 1.49 | -3.37  2.48 | -0.3 | 0.764 |
| Baseline | 0.80 | 0.05 | 0.71  0.89 | 17.3 | <.001 | 0.85 | 0.03 | 0.79  0.91 | 29.5 | <.001 | 0.97 | 0.02 | 0.93  1.00 | 58.1 | <.001 | 0.93 | 0.03 | 0.89  0.98 | 35.0 | <.001 |
| Reactivity | 1.04 | 0.09 | 0.87  1.21 | 11.8 | <.001 | 0.18 | 0.02 | 0.15  0.22 | 9.81 | <.001 | 1.87 | 0.22 | 1.43  2.30 | 8.5 | <.001 | -2.44 | 0.42 | -3.27  -1.62 | -5.8 | <.001 |
| Recovery | -1.29 | 0.10 | -1.49  -1.09 | -12.9 | <.001 | -0.23 | 0.01 | -0.25  -0.21 | -21.7 | <.001 | -10.2 | 0.52 | -11.2  -9.18 | -19.5 | <.001 | 9.07 | 0.69 | 7.71  10.4 | 13.0 | <.001 |
| **Random**  **Effects** | Var | SD | Correlation | |  | Var | SD | Correlation | |  | Var | SD | Correlation | |  | Var | SD | Correlation | |  |
| Intercept | 3.39 | 1.84 |  |  |  | 0.43 | 0.65 |  |  |  | 38.4 | 6.20 |  |  |  | 87.5 | 9.36 |  |  |  |
| Reactivity | 0.56 | 0.75 | .99 |  |  | 0.05 | 0.22 | .99 |  |  | 5.52 | 2.35 | .99 |  |  | 10.4 | 3.23 | .99 |  |  |
| Recovery | 0.40 | 0.64 | -.57 | -.54 |  | 0.01 | 0.09 | -.86 | -.79 |  | 28.3 | 5.32 | -.81 | -.84 |  | 42.2 | 6.50 | .29 | .31 |  |
| *Note:* All tests are two-sided and uncorrected. BPM = beats per minute, CI = 95% confidence interval, est = estimate, log = natural logarithm, RMSSD = root mean square of successive differences, SD = standard deviation, SE = standard error, STAI-SKD: State Trait Anxiety Inventory, Var = Variance, Model equations were as follows:  stai ~ baseline + reactivity + recovery + (1 + reactivity + recovery \| participant)  cortisol(log) ~ baseline + reactivity + recovery + (1 + reactivity + recovery \| participant)  hr ~ baseline + reactivity + recovery + (1 + reactivity + recovery \| participant)  hrv ~ baseline + reactivity + recovery + (1 + reactivity + recovery \| participant) | | | | | | | | | | | | | | | | | | | | |

**Supplementary Table 2**

*Means and standard deviations (in parentheses) for self-reported, hormonal, and autonomic stress measures at baseline, peak, and last measurement across the full sample.*

| Outcome | N | Baseline | Peak | Last |
| --- | --- | --- | --- | --- |
| STAI-SKD | 164 | 7.69 (1.88) | 10.42 (2.82) | 7.34 (2.43) |
| Cortisol (log) | 162 | 1.10 (0.51) | 1.66 (0.73) | 0.89 (0.51) |
| HR (BPM) | 162 | 73.81 (10.58) | 79.43 (12.04) | 66.52 (9.93) |
| HRV (RMSSD) | 162 | 45.62 (18.82) | 39.38 (17.00) | 58.60 (24.86) |
| *Note:* HR = heart rate (BPM = beats per minute); HRV = heart rate variability (RMSSD = root mean square of successive differences); log = natural logarithm; STAI-SKD = State-Trait Anxiety Inventory. | | | | |

*Retrospective ratings*

Two-way ANOVAs on PRS and ratings of helpfulness in managing and recovering from a hypothetical stressful event revealed significant main effects of environment (see main text) but no main effects of time and no time*environment interactions. Specifically, the main effects of time were non-significant for PRS (F(1, 160) = 0.11, p = .736), managing (F(1, 160) = 1.006, p = .317), and recovering (F(1, 160) = 1.71, p = .193). Likewise, the time*environment interactions were non-significant for PRS (F(1, 160) = 0.47, p = .495), managing (F(1, 160) = 0.106, p = .745), and recovering (F(1, 160) = 0.321, p = .572). Thus, comparing pooled nature vs. pooled indoor groups revealed significantly higher PRS scores, as well as greater perceived helpfulness in managing and recovering from a hypothetical stressor, irrespective of presentation time (pre/post). Three-sided testing to assess whether the effect of environment exceeded a smallest effect size of interest (SESOI) of d = ±0.2, revealed that the effects for PRS (d = 2.28, SE = 0.200, 95% TST CI: [0.20, 2.61], p < .001, superiority test), managing (d = 2.36, SE = 0.203, 95% TST CI: [0.20, 2.69], p < .001, superiority test), and recovering (d = 2.19, SE = 0.197, 95% TST CI: [0.20, 2.52], p < .001, superiority test) exceeded the upper SESOI, suggesting that they were superior to the SESOI (Supplementary Figure 2). Repeating these analyses with the SESOI set to d = ±0.5 or d = ±0.8 yielded similar evidence of superiority for all three outcomes. Additionally, we conducted separate TSTs for exploratory comparisons of retrospective ratings of helpfulness in preparing (nature-pre vs. indoor-pre) or recovering (nature-post vs. indoor-post) from the acute stressor. Three-sided tests revealed inconclusive evidence for helpfulness in preparing (d = 0.56, SE = 0.230, 95% TST CI: [0.11, 0.93], p = .112, superiority test), but a superior effect for helpfulness in recovering (d = 0.72, SE = 0.223, 95% TST CI: [0.20, 1.11], p = .026, superiority test) from the acute stressor. Thus, while effects for preparation remain inconclusive, recovery-related retrospective ratings suggest a practically meaningful benefit of the nature condition (Supplementary Figure 2).

**Supplementary Figure 2**. Estimated effect sizes (Cohen’s d) for retrospective restorativeness, stress management, and recovery, as well as acute stress preparation and recovery ratings. Effect sizes for the first three outcomes are based on comparisons of the pooled nature vs. indoor groups, whereas “Prepare Acute” and “Recover Acute” reflect the nature-pre vs. indoor-pre and nature-post vs. indoor-post comparisons, respectively. Confidence intervals (CI) are displayed as color-coded bands: green = 95% three-sided testing CI, blue = 95% equivalence CI, and red = 95% classic CI. The equivalence and classic CI are provided for descriptive purposes and should not be interpreted as precision estimates in the context of TST. PRS = Perceived Restorativeness Scale; TST = Three-sided testing.

*Overall stress response*

Two-way ANOVAs examining the overall stress response (quantified using area under the curve with respect to increase from baseline; AUCi) revealed no main effect of environment for STAI-SKD (F(1, 160) = 2.16, p = .143), cortisol (F(1, 158) = 0.01, p = .915), HR (F(1, 158) = 2.64, p = .106), or HRV (F(1, 158) = 1.94, p = .166). Likewise, we observed no significant main effects of time for STAI-SKD (F(1, 160) = 0.22, p = .640), cortisol (F(1, 158) = 1.06, p = .305), HR (F(1, 158) = 0.00, p = .963), or HRV (F(1, 158) = 0.03, p = .859). The time*environment interaction was non-significant for HR (F(1, 158) = 1.00, p = .318) and HRV (F(1, 158) = 0.02, p = .878), but showed a significant effect for STAI-SKD (F(1, 160) = 4.57, p = .034) and a trend-level effect for cortisol (F(1, 158) = 3.80, p = .053). Supplementary Table 3 presents post-hoc pairwise comparisons for four selected contrasts of interest (nature vs. indoor). As shown in the table, cortisol comparisons did not reach significance, while STAI-SKD revealed a significantly higher overall self-reported stress response for the nature-post vs. indoor-post comparison (after Bonferroni-Holm correction).

Additionally, we conducted three-sided testing using pooled nature vs. indoor conditions, consistent with our registered prediction of a lower overall stress response in the nature condition (irrespective of environment timing). Three-sided tests assessing whether the effect of environment exceeded our SESOI of d = ±0.2 revealed inconclusive evidence for STAI-SKD (d = -0.22, SE = 0.156, 95% TST CI: [-0.48, 0.09], p = .881, inferiority test), cortisol (d = -0.02, SE = 0.157, 90% TST CI: [-0.22, 0.18], p = .127, equivalence test), HR (d = 0.26, SE = 0.157, 95% TST CI: [-0.06, 0.52], p = .722, superiority test) and HRV (d = 0.22, SE = 0.157, 95% TST CI: [-0.09, 0.48], p = .899, superiority test). Thus, effects on the overall stress response remain inconclusive across outcomes, indicating that small negative or positive effects cannot be ruled out. However, as illustrated in Supplementary Figure 3, moderate effects (d = ±0.5) appear unlikely.

**Supplementary Table 3**

*Post-hoc pairwise comparisons of interest for overall self-reported (STAI-SKD) and hormonal (cortisol) stress responses, conducted following significant or trend-level environment*time interaction effects.*

|  | Comparison | t(df) | | p (raw) | p (adj) | d |
| --- | --- | --- | --- | --- | --- | --- |
| STAI-SKD (AUCi) | Indoor-pre vs. nature-pre | 5.26 (160) | 0.636 | | 0.959 | 0.11 |
|  | Indoor-post vs. nature-post | -28.47 (160) | 0.012 | | 0.048 | -0.55 |
|  | Indoor-pre vs. nature-post | -7.91 (160) | 0.479 | | 0.959 | -0.14 |
|  | Indoor-post vs. nature-pre | -15.30 (160) | 0.172 | | 0.516 | -0.33 |
| Cortisol (log; AUCi) | Indoor-pre vs. nature-pre | -11.01 (158) | 0.145 | | 0.581 | -0.34 |
|  | Indoor-post vs. nature-post | 9.87 (158) | 0.197 | | 0.591 | 0.27 |
|  | Indoor-pre vs. nature-post | 4.93 (158) | 0.515 | | 0.846 | 0.15 |
|  | Indoor-post vs. nature-pre | -6.08 (158) | 0.423 | | 0.846 | -0.18 |
| *Note:* Values represent t-statistics from pairwise comparisons, with both unadjusted (raw) and Bonferroni-Holm adjusted (adj) p-values; AUCi = Area under the curve with respect to increase from baseline; d = Cohen’s d (positive values indicate an effect in favor of the nature condition), STAI-SKD = State Trait Anxiety Inventory. | | | | | | |

**Supplementary Figure 3**. Estimated effect sizes (Cohen’s d) for overall stress responses across self-reported, hormonal, and autonomic outcomes. Confidence intervals (CI) are displayed as color-coded bands: green = 95% three-sided testing CI, blue = 95% equivalence CI, and red = 95% classic CI. The equivalence and classic CI are provided for descriptive purposes and should not be interpreted as precision estimates in the context of TST. AUCi = Area under the curve with respect to increase; HR = heart rate (BPM = beats per minute), HRV = heart rate variability (RMSSD = root mean square of successive differences); STAI-SKD = State Trait Anxiety Inventory; TST = Three-sided testing.

*Stress reactivity and recovery*

As reported in the main manuscript, we found no significant effects of environment on the reactivity and recovery slopes in our CPGCM for H2 and H3. Full model outputs, including model formulae, are provided in Supplementary Table 4 (reactivity) and Supplementary Table 5 (recovery). Although a clear stress reactivity and recovery response was observed (as indicated by significant fixed effects for reactivity and recovery) neither condition nor the reactivity*condition or the recovery*condition interactions were significant. We furthermore performed a sensitivity analysis using the delta scores peak-baseline (Δreactivity) and peak-final measurement (Δrecovery) for each outcome. For these analyses, we used the same participant-based peaks identified for the CPGCM to increase comparability. We then conducted separate t-tests to compare (1) Δreactivity scores between the nature-pre and indoor-pre group, and (2) Δrecovery scores between the nature-post and indoor-post groups. Supplementary Table 6 confirms the CPGM results, revealing no significant group differences in delta scores.

We further used the delta scores to perform three-sided testing. Three-sided testing assessing whether the effect of environment exceeded our SESOI of d = ±0.2 revealed inconclusive evidence for Δreactivity: STAI-SKD (d = -0.11, SE = 0.219, 90% TST CI: [-0.39, 0.18], p = .339, equivalence test), cortisol (d = -0.26, SE = 0.222, 95% TST CI: [-0.63, 0.11], p = .782, inferiority test), HR (d = 0.36, SE = 0.224, 95% TST CI: [-0.08, 0.74], p = .468, superiority test) and HRV (d = 0.24, SE = 0.223, 95% TST CI: [-0.20, 0.61], p = .869, superiority test). Likewise, we observed inconclusive evidence for Δrecovery: STAI-SKD (d = 0.15, SE = 0.225, 90% TST CI: [-0.43, 0.14], p = .403, equivalence test), cortisol (d = -0.36, SE = 0.222, 95% TST [-0.08, 0.74], p = .472, inferiority test), HR (d = 0.004, SE = 0.222, 90% TST CI: [-0.29, 0.28], p = .190, equivalence test) and HRV (d = -0.23, SE = 0.223, 95% TST CI: [0.20, 0.61], p = .878, inferiority test). Thus, effects on stress reactivity and recovery remain inconclusive across outcomes, indicating that small (and sometimes moderate) negative or positive effects cannot be ruled out (Supplementary Figure 4, panels a and b).

**Supplementary Table 4**.

*Linear mixed model results from conditional piecewise growth curve models of stress reactivity across the indoor-pre and nature-pre groups. Outcomes include self-reported (STAI-SKD), hormonal (cortisol), and autonomic (heart rate and heart rate variability) measures.*

|  | **STAI-SKD** | | | | | | | | **Cortisol (log)** | | | | | | | | | | **Heart rate (BPM)** | | | | | | | | **Heart rate variability (RMSSD)** | | | | | | | | |
| --- | --- | --- | --- | --- | --- | --- | --- | --- | --- | --- | --- | --- | --- | --- | --- | --- | --- | --- | --- | --- | --- | --- | --- | --- | --- | --- | --- | --- | --- | --- | --- | --- | --- | --- | --- |
| **Fixed Effects** | est | SE | CI | | t | | p | | est | SE | | CI | | | t | | p | | est | SE | CI | | t | | p | | est | SE | | CI | | t | | p | |
| Intercept | 3.55 | 0.53 | 2.46  4.64 | | 6.42 | | <.001 | | 0.47 | 0.10 | | 0.27  0.67 | | | 4.62 | | <.001 | | 7.16 | 2.09 | 3.04  7.16 | | 3.42 | | <.001 | | -3.39 | 2.78 | | -8.86  2.08 | | -1.22 | | .224 | |
| Baseline | 0.79 | 0.07 | 0.66  0.92 | | 12.0 | | <.001 | | 0.92 | 0.04 | | 0.83  1.00 | | | 20.3 | | <.001 | | 0.97 | 0.02 | 0.92  0.97 | | 39.5 | | <.001 | | 0.95 | 0.05 | | 0.85  1.04 | | 20.0 | | <.001 | |
| Reactivity | 1.06 | 0.16 | 0.75  1.37 | | 6.79 | | <.001 | | 0.18 | 0.04 | | 0.11  0.25 | | | 5.18 | | <.001 | | 2.11 | 0.44 | 1.24  2.10 | | 4.80 | | <.001 | | -3.04 | 0.89 | | -4.79  -1.29 | | -3.43 | | <.001 | |
| Recovery | -1.40 | 0.15 | -1.68  -1.12 | | -9.68 | | <.001 | | -0.21 | 0.01 | | -0.24  -0.19 | | | -16.1 | | <.001 | | -10.2 | 0.78 | -11.7  -10.2 | | -13.2 | | <.001 | | 8.86 | 0.92 | | 7.05  10.6 | | 9.66 | | <.001 | |
| Condition | 0.04 | 0.39 | -0.73  0.81 | | 0.09 | | .925 | | 0.15 | 0.11 | | -0.07  0.37 | | | 1.33 | | .185 | | -1.67 | 1.09 | -3.84  0.49 | | -1.54 | | .128 | | 4.03 | 2.73 | | -1.39  9.47 | | 1.47 | | .143 | |
| Reactivity*  Condition | 0.00 | 0.20 | -0.39  0.40 | | 0.01 | | .989 | | 0.03 | 0.04 | | -0.05  0.11 | | | 0.64 | | .522 | | -0.57 | 0.46 | -1.47  0.33 | | -1.25 | | .213 | | 1.16 | 1.29 | | -1.38  3.72 | | 0.90 | | .368 | |
| **Random**  **Effects** | Var | SD | Correlation | | | | | | Var | | SD | | Correlation | | | | | | Var | SD | Correlation | | | | | | Var | | SD | Correlation | | | | | |
| Intercept | 2.98 | 1.73 |  |  | |  | |  | 0.38 | | 0.61 | |  |  | |  | |  | 57.3 | 7.57 |  |  | |  | |  | 93.6 | | 9.67 |  |  | |  | |  |
| Reactivity | 0.47 | 0.68 | .98 |  | |  | |  | 0.05 | | 0.23 | | .99 |  | |  | |  | 8.26 | 2.87 | .99 |  | |  | |  | 10.9 | | 3.31 | .99 |  | |  | |  |
| Recovery | 0.51 | 0.72 | -.55 | -.43 | |  | |  | 0.00 | | 0.06 | | -.85 | -.84 | |  | |  | 33.2 | 5.76 | -.79 | -.80 | |  | |  | 36.7 | | 6.05 | .17 | .19 | |  | |  |
| Condition | 0.07 | 0.27 | -.17 | -.04 | | .87 | |  | 0.01 | | 0.10 | | .12 | .10 | | -.59 | |  | 2.32 | 1.53 | -.54 | -.53 | | -.07 | |  | 194.9 | | 13.9 | -.58 | -.56 | | .70 | |  |
| Reactivity*  Condition | 0.00 | 0.03 | -.14 | -.08 | | .44 | | .56 | 0.00 | | 0.03 | | -.79 | -.81 | | .46 | | .43 | 0.45 | 0.67 | -.55 | -.54 | | -.06 | | .99 | 22.2 | | 4.72 | -.59 | -.57 | | .69 | | .99 |
| *Note:* All tests are two-sided and uncorrected. BPM = beats per minute, CI = 95% confidence interval, est = estimate, log = natural logarithm, RMSSD = root mean square of successive differences, SD = standard deviation, SE = standard error, STAI-SKD = State Trait Anxiety Inventory, Var = Variance, Model equations were as follows:  stai ~ baseline + reactivity*condition + recovery + (1 + reactivity*condition + recovery \| participant)  cortisol(log) ~ baseline + reactivity*condition + recovery + (1 + reactivity*condition + recovery \| participant)  hr ~ baseline + reactivity*condition + recovery + (1 + reactivity*condition + recovery \| participant)  hrv ~ baseline + reactivity*condition + recovery + (1 + reactivity*condition + recovery \| participant) | | | | | | | | | | | | | | | | | | | | | | | | | | | | | | | | | | | |

**Supplementary Table 5**

*Linear mixed model results from conditional piecewise growth curve models of stress recovery across the indoor-pre and nature-pre groups. Outcomes include self-reported (STAI-SKD), hormonal (cortisol), and autonomic (heart rate and heart rate variability) measures.*

|  | **STAI-SKD** | | | | | | | | **Cortisol (log)** | | | | | | | | | | **Heart rate (BPM)** | | | | | | | | **Heart rate variability (RMSSD)** | | | | | | | | |
| --- | --- | --- | --- | --- | --- | --- | --- | --- | --- | --- | --- | --- | --- | --- | --- | --- | --- | --- | --- | --- | --- | --- | --- | --- | --- | --- | --- | --- | --- | --- | --- | --- | --- | --- | --- |
| **Fixed Effects** | est | SE | CI | | t | | p | | est | SE | | CI | | | t | | p | | est | SE | CI | | t | | p | | est | SE | | CI | | t | | p | |
| Intercept | 2.82 | 0.56 | 1.73  3.91 | | 5.06 | | <.001 | | 0.61 | 0.09 | | 0.44  0.77 | | | 7.15 | | <.001 | | 7.18 | 1.78 | 3.66  10.7 | | 4.02 | | <.001 | | 0.41 | 1.89 | | -3.31  4.13 | | 0.22 | | .828 | |
| Baseline | 0.84 | 0.06 | 0.72  0.96 | | 14.0 | | <.001 | | 0.82 | 0.03 | | 0.75  0.89 | | | 24.0 | | <.001 | | 0.95 | 0.02 | 0.91  1.00 | | 42.5 | | <.001 | | 0.91 | 0.03 | | 0.85  0.97 | | 30.9 | | <.001 | |
| Reactivity | 1.04 | 0.14 | 0.77  1.31 | | 7.67 | | <.001 | | 0.17 | 0.03 | | 0.11  0.22 | | | 6.20 | | <.001 | | 1.93 | 0.27 | 1.39  2.45 | | 7.18 | | <.001 | | -2.44 | 0.53 | | -3.47  -1.41 | | -4.64 | | <.001 | |
| Recovery | -1.23 | 0.17 | -1.58  -0.89 | | -7.08 | | <.001 | | -0.23 | 0.02 | | -0.27  -0.20 | | | -13.2 | | <.001 | | -10.3 | 0.87 | -12.0  -8.60 | | -11.9 | | <.001 | | 10.46 | 1.68 | | 7.15  13.7 | | 6.22 | | <.001 | |
| Condition | 0.37 | 0.26 | -0.15  0.89 | | 1.42 | | .160 | | 0.02 | 0.04 | | -0.06  0.11 | | | 0.57 | | .573 | | 0.01 | 0.50 | -0.98  1.01 | | 0.03 | | .977 | | 0.36 | 1.27 | | -2.17  2.90 | | 0.28 | | .776 | |
| Recovery*  Condition | 0.03 | 0.24 | -0.45  0.51 | | 0.12 | | .901 | | 0.02 | 0.02 | | -0.06  0.03 | | | -0.72 | | .473 | | 0.21 | 1.01 | -1.78  2.21 | | 0.21 | | .838 | | -2.07 | 2.16 | | -6.32  2.17 | | -0.96 | | .338 | |
| **Random**  **Effects** | Var | SD | Correlation | | | | | | Var | | SD | | Correlation | | | | | | Var | SD | Correlation | | | | | | Var | | SD | Correlation | | | | | |
| Intercept | 2.59 | 1.61 |  |  | |  | |  | 0.39 | | 0.62 | |  |  | |  | |  | 23.4 | 4.84 |  |  | |  | |  | 61.5 | | 7.84 |  |  | |  | |  |
| Reactivity | 0.69 | 0.84 | .99 |  | |  | |  | 0.05 | | 0.22 | | .99 |  | |  | |  | 3.46 | 1.86 | .99 |  | |  | |  | 7.02 | | 2.65 | .99 |  | |  | |  |
| Recovery | 0.39 | 0.63 | -.78 | -.83 | |  | |  | 0.01 | | 0.09 | | -.83 | -.78 | |  | |  | 24.1 | 4.91 | -.79 | -.85 | |  | |  | 78.7 | | 8.87 | -.21 | -.15 | |  | |  |
| Condition | 0.44 | 0.66 | .87 | .90 | | -.98 | |  | 0.02 | | 0.14 | | .98 | .98 | | -.84 | |  | 0.40 | 0.63 | .00 | .08 | | -.45 | |  | 0.00 | | 0.00 | -.36 | -.34 | | .64 | |  |
| Recovery*  Condition | 0.13 | 0.36 | .62 | .57 | | -.11 | | .24 | 0.00 | | 0.06 | | -.96 | -.97 | | .73 | | -.98 | 17.2 | 4.14 | .00 | .08 | | -.45 | | .99 | 30.9 | | 5.55 | .36 | .35 | | -.65 | | .98 |
| *Note:* All tests are two-sided and uncorrected. BPM = beats per minute, CI = 95% confidence interval, est = estimate, log = natural logarithm, RMSSD = root mean square of successive differences, SD = standard deviation, SE = standard error, STAI-SKD = State Trait Anxiety Inventory, Var = Variance, Model equations were as follows:  stai ~ baseline + reactivity + recovery*condition + (1 + reactivity + recovery*condition \| participant)  cortisol(log) ~ baseline + reactivity + recovery*condition + (1 + reactivity + recovery*condition \| participant)  hr ~ baseline + reactivity + recovery*condition + (1 + reactivity + recovery*condition \| participant)  hrv ~ baseline + reactivity + recovery*condition + (1 + reactivity + recovery*condition \| participant) | | | | | | | | | | | | | | | | | | | | | | | | | | | | | | | | | | | |

**Supplementary Table 6**

*Descriptive statistics (mean ± standard deviation) and independent-samples t-test results for stress reactivity (peak-baseline) and stress recovery (peak-last measurement) difference scores, comparing nature-pre vs. indoor-pre and nature-post vs. indoor-post groups, respectively.*

|  |  | Indoor-pre | | Nature-pre | |  |  |  |
| --- | --- | --- | --- | --- | --- | --- | --- | --- |
| Reactivity | Outcome | M | SD | M | SD | t(df) | p | d |
|  | ΔSTAI-SKD | 2.74 | 2.01 | 2.98 | 2.36 | -0.49 (81) | 0.622 | -0.11 |
|  | ΔCortisol (log) | 0.51 | 0.68 | 0.70 | 0.74 | -1.18 (80) | 0.240 | -0.26 |
|  | ΔHR (BPM) | 7.07 | 9.03 | 4.01 | 7.76 | 1.63 (79) | 0.106 | 0.36 |
|  | ΔHRV (RMSSD) | -7.41 | 11.16 | -4.49 | 13.39 | -1.07 (79) | 0.289 | 0.24 |
|  |  | Indoor-post | | Nature-post | |  |  |  |
| Recovery | Outcome | M | SD | M | SD | t(df) | p | d |
|  | ΔSTAI-SKD | 2.78 | 2.58 | 3.15 | 2.51 | -0.65 (79) | 0.516 | 0.15 |
|  | ΔCortisol (log) | 0.86 | 0.39 | 0.72 | 0.36 | 1.62 (78) | 0.108 | -0.36 |
|  | ΔHR (BPM) | 12.85 | 6.10 | 12.88 | 6.88 | -0.02 (79) | 0.984 | 0.00 |
|  | ΔHRV (RMSSD) | -19.36 | 15.22 | -16.19 | 14.20 | -1.05 (79) | 0.295 | -0.23 |
| Note: d = Cohen’s d (positive values indicate an effect in favor of the nature condition); HR = heart rate (BPM = beats per minute); HRV = heart rate variability (RMSSD = root mean square of successive differences); M = mean; SD = standard deviation; STAI-SKD = State Trait Anxiety Inventory. | | | | | | | | |

**Supplementary Figure 4**. Estimated effect sizes (Cohen’s d) for overall stress reactivity (a) and stress recovery (b) across self-reported, hormonal, and autonomic outcomes. Confidence intervals (CI) are displayed as color-coded bands: green = 95% three-sided testing CI, blue = 95% equivalence CI, and red = 95% classic CI. The equivalence and classic CI are provided for descriptive purposes and should not be interpreted as precision estimates in the context of TST. HR = heart rate (BPM = beats per minute), HRV = heart rate variability (RMSSD = root mean square of successive differences); STAI-SKD = State Trait Anxiety Inventory; TST = Three-sided testing.

*Appraisal and rumination*

As reported in the main manuscript, we found no group differences in appraisal and rumination ratings. In addition to our registered one-sided t-tests, we conducted exploratory two-sided t-tests. We observed no significant differences between the indoor-pre and nature-pre groups in primary appraisal [t(81) = -1.19, p = 0.237 (two-sided), d = -0.26, 95% CI = (-0.71, 0.19)] or secondary appraisal [t(81) = -1.08, p = .281 (two-sided), d = 0.24, 95% CI = [-0.68, 0.20)]. Similarly, indoor-post and nature-post groups did not significantly differ in negative rumination [t(79) = -0.07, p = 0.942 (two-sided), d = -0.02 95% CI = (-0.46, 0.44)] or positive rumination [t(79) = 1.23, p = 0.221 (two-sided), d = -0.27 95% CI = [-0.73, 0.19)]. Three-sided testing revealed inconclusive evidence for all outcomes: primary appraisal (d = -0.26, SE = 0.220, 95% TST CI: [-0.62, 0.17], p = .782, inferiority test), secondary appraisal (d = 0.24, SE = 0.220, 95% TST CI: [-0.20, 0.60], p = .863, superiority test), negative rumination (d = -0.02, SE = 0.222, 90% TST CI: [-0.30, 0.27], p = .205 equivalence test), and positive rumination (d = -0.27, SE = 0.223, 95% TST CI: [-0.64, 0.17], p = .742, inferiority test). Thus, effects on appraisal and rumination remain inconclusive across outcomes, indicating that small (and sometimes moderate) negative or positive effects cannot be ruled out (Supplementary Figure 5 panel a and b).

**Supplementary Figure 5**. Estimated effect sizes (Cohen’s d) for appraisal (a) and rumination (b). Confidence intervals (CI) are displayed as color-coded bands: green = 95% three-sided testing CI, blue = 95% equivalence CI, and red = 95% classic CI. The equivalence and classic CI are provided for descriptive purposes and should not be interpreted as precision estimates in the context of TST. TST = Three-sided testing.

**Supplementary Figure 6**. Forest plot of estimated effect sizes (Cohen’s d) across all preregistered hypotheses related to immediate stress responses, grouped according to the corresponding hypothesis. Confidence intervals (CI) are displayed as color-coded bands: green = 95% three-sided testing CI, blue = 95% equivalence CI, and red = 95% classic CI. The equivalence and classic CI are provided for descriptive purposes and should not be interpreted as precision estimates in the context of TST. HR = heart rate (BPM = beats per minute); HRV = heart rate variability (RMSSD = root mean square of successive differences); STAI-SKD = State Trait Anxiety Inventory; TST = Three-sided testing.

*Association of retrospective ratings and immediate stress responses*

To investigate whether retrospective ratings of the environments were associated with immediate stress responses, we conducted additional correlation analyses. Specifically, we correlated ratings of perceived restorativeness (PRS) with overall stress responses (AUCi), and with difference (delta) scores indexing reactivity (peak-baseline) and recovery (peak-last measurement) for all immediate outcome measures (STAI-SKD, cortisol, HR, HRV) across all four groups. We also correlated retrospective ratings of helpfulness in preparing for the stressor with all immediate outcomes in groups 1 and 2, which were exposed to the environments before the stressor. Furthermore, we correlated retrospective ratings of helpfulness in recovering from the stressor with all immediate outcomes in groups 3 and 4, which were exposed to the environments after the stressor. For each association, we assessed Pearson correlation coefficients and distance correlation coefficients (in parentheses) to capture linear and non-linear relationships, respectively (Supplementary Table 7). Note that distance correlations range from 0 to 1 and quantify the overall strength of an association, including non-linear patterns (Székely et al., 2007). However, they do not provide information about the direction of an effect (i.e., positive or negative). As shown in Supplementary Table 7, both linear and non-linear associations were negligible to small in magnitude. Only one association reached significance before correcting for multiple comparisons. Overall, the results indicate that immediate stress responses were not reflected in retrospective assessments of the environments, underscoring the apparent dissociation between group effects on retrospective ratings and on immediate stress measures.

**Supplementary Table 7**

*Pearson correlations and distance correlations (in parentheses) between retrospective ratings (perceived restorativeness, helpfulness in preparing for the stressor, and helpfulness in recovering from the stressor) and immediate acute stress responses across self-reported (STAI-SKD), hormonal (cortisol), and autonomic (heart rate and heart rate variability) measures.*

|  | Outcome | PRS  (all groups) | Prepare acute  (groups 1 & 2) | Recover acute  (groups 3 & 4) |
| --- | --- | --- | --- | --- |
| STAI-SKD | AUCi | .09  (.18) | -.18  (.21) | .09  (.21) |
|  | Δreactivity | .12  (.17) | -.10  (.19) | .12  (.19) |
|  | Δrecovery | .07  (.12) | .02  (.10) | .25*  (.33) |
| Cortisol (log) | AUCi | -.03  (.12) | -.13  (.18) | -.05  (.18) |
|  | Δreactivity | -.06  (.14) | -.11  (.15) | -.06  (.18) |
|  | Δrecovery | -.10  (.13) | -.04  (.14) | .00  (.14) |
| HR (BPM) | AUCi | .00  (.14) | -.08  (.14) | -.02  (.14) |
|  | Δreactivity | .00  (.14) | -.02  (.16) | .04  (.13) |
|  | Δrecovery | -.01  (.12) | .09  (.18) | .06  (.15) |
| HRV (RMSSD) | AUCi | .03  (.10) | .10  (.16) | .15  (.23) |
|  | Δreactivity | .10  (.13) | .14  (.17) | .20`  (.27) |
|  | Δrecovery | .17  (.17) | .00  (.11) | .04  (.17) |
| Note: Numbers represent Pearson and distance correlations (in parentheses). AUCi = Area under the curve with respect to increase from baseline; HR = heart rate (BPM = beats per minute); HRV = heart rate variability (RMSSD = root mean square of successive differences); PRS = Perceived Restorativeness Scale; STAI-SKD = State Trait Anxiety Inventory; ` p = .075 (uncorrected); * p < .05 (uncorrected). | | | | |

*Association of nature connectedness, lifetime urban exposure, and immediate stress responses*

To address potential influences of interindividual differences in participants’ preferences for nature or prior environmental exposure, we conducted non-registered exploratory analyses examining whether nature connectedness, lifetime urban exposure and their interactions with environmental condition (nature vs. indoor) predicted immediate stress-related outcomes. Specifically, we ran a series of regression analyses using environment (nature vs. indoor), nature connectedness (NCI; Richardson et al., 2019), lifetime urban exposure (Lederbogen et al., 2011), as well as the interaction terms “nature connectedness*environment” and “lifetime urban exposure*environment”, as predictors of overall stress response (AUCi), reactivity (peak-baseline), and recovery (peak-last measurement) across all immediate stress-related outcomes (STAI-SKD, cortisol, HR, HRV), resulting in 12 separate models (Supplementary Table 8). The interaction terms were included to test whether individual differences in nature connectedness or lifetime urban exposure moderated the effects of environments on stress-related outcomes. None of the interaction effects significantly predicted any of the outcomes. There was, however, a main effect of lifetime urban exposure on all cortisol outcomes, indicating that higher lifetime urban exposure was associated with a stronger overall cortisol response, as well as more pronounced reactivity and recovery (with small effect sizes). This is consistent with prior work linking urbanicity to increased neural responses to acute social evaluative stress in the anterior cingulate cortex and amygdala (Lederbogen et al., 2011). Notably, there was also a significant main effect of lifetime urban exposure on HRV recovery which did not survive multiple comparison correction (Bonferroni-Holm corrected across the three tests of lifetime urban exposure in HRV-related outcomes, p_adj_ = .069). Overall, these results suggest that nature connectedness and a proxy for prior exposure to different environments did not meaningfully moderate emotional or physiological responses to the environments presented.

**Supplementary Table 8**

*Linear regression results using nature connectedness, lifetime urban exposure and their interaction with environment (nature vs. urban) as predictors for overall immediate stress responses, stress reactivity, and stress recovery across stress related outcomes (STAI-SKD, cortisol, HR, HRV).*

|  |  | β | est | SE | CI | t | p |
| --- | --- | --- | --- | --- | --- | --- | --- |
| STAI  (AUCi) | Intercept |  | 14.51 | 5.59 | 3.37, 25.44 | 2.58 | .011 |
|  | Env | 0.19 | 9.86 | 7.96 | -5.85, 25.58 | 1.24 | .216 |
|  | NCI | 0.01 | 0.01 | 0.23 | -0.45, 0.47 | 0.05 | .962 |
|  | Urbanicity | -0.06 | -4.03 | 7.86 | -19.55, 11.49 | -0.51 | .609 |
|  | NCI*Env | -0.20 | -0.42 | 0.33 | -1.08, 0.24 | -1.26 | .208 |
|  | Urbanicity*Env | -0.15 | -10.82 | 11.22 | -32.97, 11.33 | -0.97 | .336 |
| STAI  (Δreactivity) | Intercept |  | 2.39 | 0.26 | 1.87, 2.90 | 9.12 | <.001 |
|  | Env | 0.28 | 0.66 | 0.37 | -0.08, 1.39 | 1.76 | .079 |
|  | NCI | 0.09 | 0.01 | 0.01 | -0.01, 0.03 | 0.81 | .421 |
|  | Urbanicity | -0.02 | -0.08 | 0.37 | -0.80, 0.65 | -0.21 | .835 |
|  | NCI*Env | -0.16 | -0.02 | 0.02 | -0.04, 0.02 | -0.98 | .327 |
|  | Urbanicity*Env | -0.10 | -0.34 | 0.52 | -1.38, 0.69 | -0.65 | .515 |
| STAI  (Δrecovery) | Intercept |  | 2.90 | 0.28 | 2.36, 3.45 | 10.48 | <.001 |
|  | Env | 0.14 | 0.36 | 0.39 | -0.42, 1.14 | 0.91 | .365 |
|  | NCI | 0.11 | 0.01 | 0.01 | -0.01, 0.03 | 0.99 | .326 |
|  | Urbanicity | 0.04 | 0.15 | 0.39 | -0.62, 0.92 | 0.39 | .700 |
|  | NCI*Env | -0.0003 | -0.003 | 0.02 | -0.03, 0.03 | -0.02 | .986 |
|  | Urbanicity*Env | -0.005 | -0.001 | 0.56 | -1.10, 1.09 | -0.01 | .993 |
| Cortisol  (log; AUCi) | Intercept |  | 6.17 | 3.78 | -1.29, 13.63 | 1.63 | .104 |
|  | Env | 0.03 | 1.06 | 5.33 | -9.49, 11.60 | 0.19 | .843 |
|  | NCI | -0.11 | -0.16 | 0.16 | -0.48, 0.15 | -1.02 | .309 |
|  | Urbanicity | 0.31 | 14.75 | 5.28 | 4.33, 25.17 | 2.79 | .005 |
|  | NCI*Env | 0.03 | 0.05 | 0.23 | -0.40, 0.49 | 0.20 | .839 |
|  | Urbanicity*Env | -0.22 | -10.64 | 7.49 | -25.44, 4.16 | -1.42 | .157 |
| Cortisol  (log; Δreactivity) | Intercept |  | 0.57 | 0.08 | 0.41, 0.73 | 6.91 | <.001 |
|  | Env | -0.02 | -0.01 | 0.12 | -0.24, 0.22 | -0.12 | .907 |
|  | NCI | -0.08 | -0.002 | 0.003 | -0.01, 0.004 | -0.73 | .469 |
|  | Urbanicity | 0.33 | 0.34 | 0.11 | 0.12, 0.57 | 3.00 | .003 |
|  | NCI*Env | -0.03 | -0.001 | 0.005 | -0.01, 0.01 | -0.20 | .841 |
|  | Urbanicity*Env | -0.22 | -0.23 | 0.16 | -0.55, 0.09 | -1.43 | .153 |
| Cortisol  (log; Δrecovery) | Intercept |  | 0.79 | 0.04 | 0.71, 0.86 | 19.36 | <.001 |
|  | Env | -0.13 | -0.04 | 0.06 | -0.16, 0.07 | -0.83 | .410 |
|  | NCI | -0.02 | -0.0003 | 0.002 | -0.004, 0.003 | -0.21 | .832 |
|  | Urbanicity | 0.34 | 0.18 | 0.06 | 0.07, 0.29 | 3.17 | .002 |
|  | NCI*Env | -0.17 | -0.003 | 0.002 | -0.01, 0.002 | -1.08 | .283 |
|  | Urbanicity*Env | -0.24 | -0.13 | 0.08 | -0.28, 0.03 | -1.55 | .123 |
| HR  (AUCi) | Intercept |  | 24.86 | 14.31 | -3.41, 53.14 | 1.74 | .084 |
|  | Env | -0.25 | -31.89 | 20.40 | -72.19, 8.42 | -1.56 | .120 |
|  | NCI | -0.13 | -0.69 | 0.61 | -1.89, 0.51 | -1.14 | .258 |
|  | Urbanicity | 0.07 | 13.52 | 20.14 | -26.28, 53.31 | 0.67 | .503 |
|  | NCI*Env | 0.19 | 1.04 | 0.86 | -0.67, 2.74 | 1.20 | .231 |
|  | Urbanicity*Env | 0.02 | 3.13 | 28.79 | -53.73, 59.99 | 0.11 | .914 |
| HR  (Δreactivity) | Intercept |  | 6.52 | 0.83 | 4.88, 8.15 | 7.88 | <.001 |
|  | Env | -0.23 | -1.73 | 1.18 | -4.07, 0.59 | -1.47 | .143 |
|  | NCI | -0.10 | -0.03 | 0.03 | -0.09, 0.04 | -0.86 | .394 |
|  | Urbanicity | 0.05 | 0.56 | 1.17 | -1.74, 2.86 | 0.48 | .633 |
|  | NCI*Env | 0.21 | 0.07 | 0.05 | -0.03, 0.16 | 1.32 | .189 |
|  | Urbanicity*Env | 0.01 | 0.13 | 1.66 | -3.16, 3.42 | 0.08 | .938 |
| HR  (Δrecovery) | Intercept |  | 13.53 | 0.78 | 12.00, 15.06 | 17.45 | <.001 |
|  | Env | -0.18 | -1.22 | 1.11 | -3.40, 0.96 | -1.10 | .272 |
|  | NCI | -0.02 | -0.01 | 0.03 | -0.07, 0.06 | -0.15 | .879 |
|  | Urbanicity | -0.04 | -0.35 | 1.09 | -2.51, 1.80 | -0.32 | .748 |
|  | NCI*Env | 0.10 | 0.03 | 0.05 | -0.06, 0.12 | 0.63 | .526 |
|  | Urbanicity*Env | 0.01 | 0.11 | 1.56 | -2.97, 3.19 | 0.07 | .946 |
| HRV  (AUCi) | Intercept |  | 28.93 | 37.54 | --45.25, 103.08 | 0.77 | .442 |
|  | Env | 0.21 | 72.05 | 53.51 | -33.66, 177.75 | 1.35 | .180 |
|  | NCI | 0.15 | 2.09 | 1.59 | -1.07, 5.25 | 1.31 | .194 |
|  | Urbanicity | -0.09 | -41.39 | 52.83 | -145.76, 62.97 | -0.78 | .434 |
|  | NCI*Env | -0.30 | -4.33 | 2.26 | -8.79, 0.13 | -1.92 | .057 |
|  | Urbanicity*Env | 0.07 | 35.42 | 75.49 | -113.69, 184.54 | 0.47 | .639 |
| HRV  (Δreactivity) | Intercept |  | -7.94 | 1.28 | -10.47, -5.40 | -6.19 | <.001 |
|  | Env | 0.28 | 3.31 | 1.83 | -0.31, 6.92 | 1.81 | .073 |
|  | NCI | 0.06 | 0.03 | 0.05 | -0.08, 0.14 | 0.54 | .589 |
|  | Urbanicity | -0.07 | -1.14 | 1.81 | -4.71, 2.42 | -0.63 | .527 |
|  | NCI*Env | -0.25 | -0.12 | 0.98 | -0.27, 0.03 | -1.55 | .122 |
|  | Urbanicity*Env | 0.07 | 1.12 | 2.58 | -3.97, 6.22 | 0.44 | .663 |
| HRV  (Δrecovery) | Intercept |  | -20.76 | 1.77 | -24.25, -17.27 | -11.75 | <.001 |
|  | Env | 0.19 | 3.04 | 2.52 | -1.93, 8.02 | 1.21 | .228 |
|  | NCI | -0.11 | -0.08 | 0.08 | -0.22, 0.07 | -1.01 | .315 |
|  | Urbanicity | 0.25 | 5.73 | 2.48 | 0.81, 10.64 | 2.30 | .023 |
|  | NCI*Env | 0.14 | 0.09 | 0.11 | -0.12, 0.30 | 0.88 | .382 |
|  | Urbanicity*Env | -0.24 | -5.42 | 3.55 | -12.44, 1.60 | -1.53 | .129 |
| Note: β = standardized beta, AUCi = Area under the curve with respect to increase from baseline, est = estimate, Env = environment, NCI = Nature connectedness index, Urbanicity = Lifetime urban exposure; CI = 95% confidence interval (estimate), SE = standard error (estimate) | | | | | | | |

**References**

Adiasto, K., Beckers, D. G. J., Van Hooff, M. L. M., Roelofs, K., & Geurts, S. A. E. (2022). Music listening and stress recovery in healthy individuals: A systematic review with meta-analysis of experimental studies. *PLOS ONE*, *17*(6), e0270031. https://doi.org/10.1371/journal.pone.0270031

Aikoh, T., Homma, R., & Abe, Y. (2023). Comparing conventional manual measurement of the green view index with modern automatic methods using google street view and semantic segmentation. *Urban Forestry & Urban Greening*, *80*, 127845. https://doi.org/10.1016/j.ufug.2023.127845

Annerstedt, M., Jönsson, P., Wallergård, M., Johansson, G., Karlson, B., Grahn, P., Hansen, Å. M., & Währborg, P. (2013). Inducing physiological stress recovery with sounds of nature in a virtual reality forest—Results from a pilot study. *Physiology & Behavior*, *118*, 240–250. https://doi.org/10.1016/j.physbeh.2013.05.023

Aoki, Y. (1991). Evaluation methods for landscapes with greenery. *Landscape Research*, *16*(3), 3–6. https://doi.org/10.1080/01426399108706344

Berto, R. (2005). Exposure to restorative environments helps restore attentional capacity. *Journal of Environmental Psychology*, *25*(3), 249–259. https://doi.org/10.1016/j.jenvp.2005.07.001

De Witte, M., Spruit, A., Van Hooren, S., Moonen, X., & Stams, G.-J. (2020). Effects of music interventions on stress-related outcomes: A systematic review and two meta-analyses. *Health Psychology Review*, *14*(2), 294–324. https://doi.org/10.1080/17437199.2019.1627897

Edwards, S. L., Rapee, R. M., & Franklin, J. (2003). Postevent rumination and recall bias for a social performance event in high and low socially anxious individuals. *Cognitive Therapy and Research*, *27*(6), 603–617. https://doi.org/https://doi.org/10.1023/A:1026395526858

Englert, C., Bertrams, A., & Dickhäuser, O. (2011). Entwicklung der Fünf-Item-Kurzskala STAI-SKD zur Messung von Zustandsangst. *Zeitschrift für Gesundheitspsychologie*, *19*(4), 173–180. https://doi.org/10.1026/0943-8149/a000049

Faul, F., Erdfelder, E., Buchner, A., & Lang, A.-G. (2009). Statistical power analyses using G*Power 3.1: Tests for correlation and regression analyses. *Behavior Research Methods*, *41*(4), 1149–1160. https://doi.org/10.3758/BRM.41.4.1149

Gaab, J., Rohleder, N., Nater, U. M., & Ehlert, U. (2005). Psychological determinants of the cortisol stress response: The role of anticipatory cognitive appraisal. *Psychoneuroendocrinology*, *30*(6), 599–610. https://doi.org/10.1016/j.psyneuen.2005.02.001

Grimm, K. J., Ram, N., & Estabrook, R. (2016). *Growth modeling: Structural equation and multilevel modeling approaches*. Guilford Publications.

Hartig, T., Korpela, K., Evans, G. W., & Gärling, T. (1997). A measure of restorative quality in environments. *Scandinavian Housing and Planning Research*, *14*(4), 175–194. https://doi.org/10.1080/02815739708730435

Kaplan, S. (1995). The restorative benefits of nature: Toward an integrative framework. *Journal of Environmental Psychology*, *15*(3), 169–182. https://doi.org/10.1016/0272-4944(95)90001-2

Kirschbaum, C., Klauer, T., Filipp, S.-H., & Hellhammer, D. H. (1995). Sex-Specific Effects of Social Support on Cortisol and Subjective Responses to Acute Psychological Stress: *Psychosomatic Medicine*, *57*(1), 23–31. https://doi.org/10.1097/00006842-199501000-00004

Lederbogen, F., Kirsch, P., Haddad, L., Streit, F., Tost, H., Schuch, P., Wüst, S., Pruessner, J. C., Rietschel, M., Deuschle, M., & Meyer-Lindenberg, A. (2011). City living and urban upbringing affect neural social stress processing in humans. *Nature*, *474*(7352), 498–501. https://doi.org/10.1038/nature10190

Liszio, S., Graf, L., & Masuch, M. (2018). The relaxing effect of virtual nature: Immersive technology provides relief in acute stress situations. *Annual Review of Cybertherapy and Telemedicine*, *16*, 87–93.

Lopez-Duran, N. L., Mayer, S. E., & Abelson, J. L. (2014). Modeling neuroendocrine stress reactivity in salivary cortisol: Adjusting for peak latency variability. *Stress*, *17*(4), 285–295. https://doi.org/10.3109/10253890.2014.915517

Mirman, D. (2017). *Growth curve analysis and visualization using R*. Chapman and Hall/CRC.

Pinheiro, J., Bates, D., & R Core Team. (2025). *nlme: Linear and Nonlinear Mixed Effects Models* [Computer software]. https://CRAN.R-project.org/package=nlme

Richardson, M., Hunt, A., Hinds, J., Bragg, R., Fido, D., Petronzi, D., Barbett, L., Clitherow, T., & White, M. (2019). A measure of nature connectedness for children and adults: Validation, performance, and insights. *Sustainability*, *11*(12), 3250. https://doi.org/10.3390/su11123250

Székely, G. J., Rizzo, M. L., & Bakirov, N. K. (2007). Measuring and testing dependence by correlation of distances. *The Annals of Statistics*, *35*(6). https://doi.org/10.1214/009053607000000505

Thoma, M. V., La Marca, R., Brönnimann, R., Finkel, L., Ehlert, U., & Nater, U. M. (2013). The effect of music on the human stress response. *PLoS ONE*, *8*(8), e70156. https://doi.org/10.1371/journal.pone.0070156

Villanueva-Rivera, L. J., Pijanowski, B. C., Doucette, J., & Pekin, B. (2011). A primer of acoustic analysis for landscape ecologists. *Landscape Ecology*, *26*(9), 1233–1246. https://doi.org/10.1007/s10980-011-9636-9

Xie, E., Wang, W., Yu, Z., Anandkumar, A., Alvarez, J. M., & Luo, P. (2021). SegFormer: Simple and Efficient Design for Semantic Segmentation with Transformers. In M. Ranzato, A. Beygelzimer, Y. Dauphin, P. S. Liang, & J. W. Vaughan (Eds.), *Advances in Neural Information Processing Systems* (Vol. 34, pp. 12077–12090).

Zhou, B., Zhao, H., Puig, X., Fidler, S., Barriuso, A., & Torralba, A. (2017). Scene Parsing through ADE20K Dataset. *2017 IEEE Conference on Computer Vision and Pattern Recognition (CVPR)*, 5122–5130. https://doi.org/10.1109/CVPR.2017.544
